# Intravenous versus oral iron for anaemia among pregnant women in Nigeria (IVON): an open-label, randomised controlled trial

**DOI:** 10.1016/S2214-109X(24)00239-0

**Published:** 2024-09-18

**Authors:** Bosede B Afolabi, Ochuwa A Babah, Titilope A Adeyemo, Mobolanle Balogun, Aduragbemi Banke-Thomas, Ajibola I Abioye, Opeyemi R Akinajo, Hadiza S Galadanci, Rachel A Quao, Hameed Adelabu, Nadia A Sam-Agudu, Victoria O Adaramoye, Abdulazeez Abubakar, Bolanle Banigbe, Gbenga Olorunfemi, Lenka Beňová, Elin C Larsson, Kristi S Annerstedt, Claudia Hanson, Jim Thornton, IVON Trial Investigators, Olufemi Motunolani Omololu, Hakeem Abayomi Agbetoba, Mercy Alokha, Abimbola Seun Oyinlade, Oluwatosin Ogunsanwo, Sule Abdullahi Gaya, Halima Ibrahim-Bello, Samuel Olusegun Spaine, Yusuf Saleh, Khadija Adam, Sabo Umar-Suleiman

**Affiliations:** a Department of Obstetrics and Gynaecology, Faculty of Clinical Sciences, College of Medicine, University of Lagos, Lagos State, Nigeria; b Department of Haematology and Blood Transfusion, Faculty of Clinical Sciences, College of Medicine, University of Lagos, Lagos State, Nigeria; c Department of Community Health and Primary Care, Faculty of Clinical Sciences, College of Medicine, University of Lagos, Lagos State, Nigeria; d Centre for Clinical Trials and Implementation Science, College of Medicine, University of Lagos, Lagos State, Nigeria; e Department of Global Public Health, Karolinska Institutet, Stockholm, Sweden; f Maternal Adolescent Reproductive and Child Health Centre, London School of Hygiene & Tropical Medicine, London, UK; g Department of Global Health and Population, Harvard T H Chan School of Public Health, Boston, MA, USA; h Department of Obstetrics and Gynaecology, Aminu Kano Teaching Hospital, Kano, Nigeria; i Global Pediatrics Program and Division of Infectious Diseases, Department of Pediatrics, University of Minnesota Medical School, Minneapolis, MN, USA; j Resolve to Save Lives, New York City, NY, USA; k Division of Epidemiology and Biostatistics, School of Public Health, University of Witwatersrand, Johannesburg, South Africa; l Department of Public Health, Institute of Tropical Medicine, Antwerp, Belgium; m School of Medicine, University of Nottingham, Nottingham, UK

## Abstract

**Background:**

Oral iron for anaemia in pregnancy is often not well tolerated, with poor adherence. Iron administered intravenously might address these tolerance and adherence issues. We investigated the effectiveness and safety of intravenous ferric carboxymaltose versus oral ferrous sulphate on anaemia and iron deficiency among pregnant women in Nigeria.

**Methods:**

We did a multicentre, open-label, parallel, randomised controlled trial of pregnant women (aged 15–49 years) with haemoglobin (Hb) concentrations of less than 10 g/dL at 20–32 weeks’ gestation from 11 primary, secondary, or tertiary health facilities in Nigeria (five in Lagos and six in Kano). Exclusion criteria included vaginal bleeding, blood transfusion or major surgery within the past 3 months, symptomatic anaemia, anaemia known to be unrelated to iron deficiency, clinically confirmed malabsorption syndrome, previous hypersensitivity to any form of iron, pre-existing maternal depression or other major psychiatric illness, immune-related diseases, such as systemic lupus erythematosus or rheumatoid arthritis, or severe allergic reactions. Participants were randomly assigned (1:1) by nurses and doctors using a web-based randomisation service to either receive a single dose of intravenous ferric carboxymaltose (20 mg/kg to a maximum of 1000 mg) or oral ferrous sulphate (200 mg; 65 mg elemental iron) three times daily until 6 weeks postpartum. The study was primarily unmasked. Primary outcomes were maternal anaemia (Hb <11 g/dL) at 36 weeks’ gestation and preterm birth at before 37 weeks’ gestation, with analysis by intention to treat in participants with available data. This study was registered at the ISRCTN registry on Dec 10, 2020 (ISRCTN63484804) and on ClinicalTrials.gov (NCT04976179) on April 7, 2021.

**Findings:**

Between Aug 10, 2021, and Dec 15, 2022, 13 724 pregnant women were screened for eligibility. 12 668 were excluded due to ineligibility for inclusion, and 1056 provided consent to participate and were randomly assigned to either the intravenous or oral administration groups. 527 were assigned to the intravenous ferric carboxymaltose group and 529 were assigned to the oral ferrous sulphate group. 518 in the intravenous group were assessed at 36 weeks’ gestational age and after 518 deliveries, and 511 completed the 6 weeks postpartum visit. 513 in the oral ferrous sulphate group were assessed at 36 weeks’ gestational age and after 512 deliveries, and 501 completed the 6 weeks postpartum visit. No significant difference was found in anaemia at 36 weeks (299 [58%] of 517 in the intravenous group *vs* 305 [61%] of 503 in the oral group; risk ratio 0·95, 95% CI 0·85–1·06; p=0·36), nor in preterm birth (73 [14%] of 518 *vs* 77 [15%] of 513; 0·94, 0·70–1·26; p=0·66). There were no significant differences in adverse events. The most common adverse events were diarrhoea (in six participants) and vomiting (in three participants) in the oral group and fatigue (in two participants) and headache (in two participants) in the intravenous group.

**Interpretation:**

Although the effect on overall anaemia did not differ, intravenous iron reduced the prevalence of iron deficiency to a greater extent than oral iron and was considered to be safe. We recommend that intravenous iron be considered for anaemic pregnant women in Nigeria and similar settings.

**Funding:**

Bill & Melinda Gates Foundation.

## Introduction

Anaemia, defined as a haemoglobin (Hb) concentration of less than 11 g/dL, affects 37% of pregnant women globally, but disproportionately affects 46% of pregnant women in Africa and 48% in southeast Asia.^1^ In Nigeria, the most populous country in Africa, the prevalence of anaemia in pregnancy is 56%,^1^ and 25–46% of these women have concurrent iron deficiency.^2^ Irrespective of the cause, anaemia in pregnancy is associated with increased mortality from obstetric haemorrhage, maternal infections and depression, and infant prematurity and low birthweight.^3^, ^4^

**Research in context**

**Evidence before this study**

We searched PubMed for articles published in English using the terms “intravenous iron” and “pregnancy”. All papers published from database inception to Oct 27, 2023, were considered. We found 21 randomised controlled trials from eight systematic reviews, which compared intravenous iron with oral iron during pregnancy. Available evidence found intravenous iron to be safe and more effective than oral iron for treating anaemia or iron deficiency anaemia. A 2019 systematic review of randomised controlled trials on intravenous versus oral iron for iron deficiency anaemia in pregnancy included 20 studies. 13 of the trials examined intravenous sucrose and three specifically examined the effect of intravenous ferric carboxymaltose versus oral iron. Only eight of the trials assessed clinical outcomes and none of them found any difference in effect on substantive clinical outcomes such as rates of blood transfusion, birthweight, preterm birth, or neonatal morbidity. We independently reviewed the three trials involving ferric carboxymaltose; all of them were conducted in upper-middle-income and high-income countries, including South Korea, Russia, Sweden, Switzerland, Australia, and Türkiye. One of them was a subgroup post hoc analysis of the second one and did not find significant differences in one of the countries (South Korea). The remaining two both found ferric carboxymaltose more effective for reducing the risk of anaemia and for correcting haemoglobin and ferritin concentrations. They reported on rate of blood transfusion, maternal quality of life, and a few neonatal parameters such as Apgar score and birthweight but did not examine any other maternal or neonatal clinical outcomes. Neither of the two studies found any significant differences in the clinical outcomes they examined except for some parameters of quality of life. Another 2019 systematic review included 18 studies on antenatal or postpartum women and found a significantly higher rise in serum ferritin with intravenous iron compared with oral iron. All included articles evaluated laboratory outcomes (haemoglobin concentration, ferritin, and iron levels). Six of the 18 studies evaluated clinical outcomes, largely neonate-related. Only two articles examined intravenous iron effect on infant prematurity. Reported clinical outcomes (gestational age at delivery, fetal birthweight, and Apgar scores) were not statistically different in the intravenous versus oral groups. A recent study conducted in Malawi (east Africa) found no effect of ferric carboxymaltose on anaemia but found an effect on iron deficiency anaemia. The Malawi study did not examine the effect on depression or any postnatal clinical outcomes.

**Added value of this study**

Our study was conducted in Nigeria, which has the highest annual number of pregnant women in Africa and a relatively high prevalence of anaemia among women of reproductive age. To the best of our knowledge, our study is the largest randomised controlled trial comparing the effectiveness of intravenous iron versus oral ferrous sulphate among pregnant women, the second in Africa, and the first in west Africa. We also evaluated the effect of ferric carboxymaltose treatment on postnatal maternal outcomes, including maternal depression, (which contributes substantially to maternal morbidity), and breastfeeding and immunisation practices. Our study found that intravenous iron was more effective than oral iron in increasing mean haemoglobin concentrations at 4 weeks post-treatment, and nearly eliminated iron deficiency among treated women at 36 weeks’ gestation.

**Implications of all the available evidence**

Our trial provides further evidence to support existing data that, compared with oral iron, intravenous iron is more effective at reducing anaemia in those with anaemia deemed at high risk of iron deficiency or iron deficiency anaemia. However, there is no evidence of an effect on anaemia in general. Intravenous iron should therefore be considered for women with confirmed iron deficiency or iron deficiency anaemia, or those at high risk of iron deficiency or iron deficiency anaemia. Further research on the degree of anaemia for which intravenous iron treatment (*vs* blood transfusion) will be effective is required. Available evidence also makes a case for affordable point-of-care rapid diagnostic tests to screen for iron deficiency in high-burden, low-resourced settings.

WHO recommends an oral daily dose of 120 mg elemental iron for the treatment of anaemia in pregnancy,^5^ but this therapy has been associated with vomiting, diarrhoea, and abdominal pain, which limit adherence.^6^, ^7^ Additionally, repeated prescriptions in resource-limited settings where women delay or infrequently seek antenatal care is a barrier to completing treatment as the women cannot obtain their drugs when required without visiting the health facility.^8^ Intravenous iron formulations are an alternative, because they have been found to be effective in raising Hb concentrations at various timepoints and in reducing the risk of iron deficiency anaemia when compared with oral iron.^9^, ^10^ However, some formulations (eg, high molecular weight dextrans) have been associated with anaphylactic reactions.^11^ Other intravenous formulations, such as iron sucrose and iron polymaltose, require multiple treatment doses and prolonged infusion times, respectively. Newer intravenous iron preparations administered as a single rapid infusion are increasingly used.^7^ Intravenous ferric carboxymaltose is one such preparation; it is administered as a 20 mg/kg infusion to a maximum of 1000 mg over 15 min and has no identified safety issues except transient hypophosphataemia.^12^

In a 2019 systematic review of randomised controlled trials of intravenous versus oral iron for anaemia in pregnancy, all the trials had a sample size of 252 women or fewer. Furthermore, the studies reported important clinical outcomes, such as blood transfusion, birthweight, and preterm delivery, but did not include others, such as maternal depression, haemorrhage, sepsis, breastfeeding, or immunisation practices. Additionally, 14 of the 20 trials focused on iron sucrose, and only three examined ferric carboxymaltose.^13^ Most of the previous randomised controlled trials of intravenous versus oral iron during pregnancy were conducted in North America, Europe, and Asia,^13^, ^14^ with only one in Africa.^15^ The African trial compared ferric carboxymaltose with 60 mg elemental oral iron twice daily in 862 pregnant Malawian women in the second trimester. The study found a significantly higher increase in Hb concentration at 4 weeks after treatment and a lower prevalence of iron deficiency anaemia at 36 weeks’ gestation, at delivery, and at 4 weeks postpartum. However, there was no difference in the prevalence of anaemia at 36 weeks’ gestation, nor for any adverse perinatal outcomes.^15^ The trial did not measure maternal depression or other outcomes such as immunisation or breastfeeding practices.

We aimed to investigate the comparative effectiveness and safety of intravenous iron (ferric carboxymaltose) versus oral iron (ferrous sulphate) in pregnant Nigerian women for reducing the prevalence of anaemia and iron deficiency at 36 weeks’ gestation. We also aimed to assess the effect on the incidence of preterm birth and other clinical outcomes, such as maternal depression, infections, immunisation, and breastfeeding practices.

## Methods

### Study design

This study was a multicentre, open-label, parallel, randomised controlled trial with a type 1 hybrid effectiveness–implementation study. The protocol details were published previously,^16^ and this Article focuses on the effectiveness trial results. The trial was conducted in the two most populous states in Nigeria, which are readily accessible, lie in different geographical zones (Lagos in the south and Kano in the north), and have diverse characteristics in terms of culture, religion, and inequalities in health care. Five health facilities were selected in Lagos (two primary, two secondary, and one tertiary) and six in Kano State (three primary, two secondary, and one tertiary), all with high antenatal patient loads (appendix 1 p 1). One facility in Kano was replaced 3 months after commencing enrolment due to service deterioration and, consequently, difficulty with participant clinic attendance and follow-up.

Ethical approval was obtained from National Health Research Ethics Committee (NHREC/01/01/2007- 04/02/2021), Lagos University Teaching Hospital Health Research Ethics Committee (ADM/DCST/HREC/APP/3971), Kano State Ministry of Health (MOH/Off/797/T.1/2102), and Aminu Kano Teaching Hospital (NHREC/28/01/2020/AKTH/EC/2955). Permission to conduct research in the secondary and primary health facilities in Lagos State was obtained from the Lagos State Health Service Commission (LSHSC/2222/VOLIII) and Lagos State Primary Health Care Board (LS/PHCB/MS/1128/VOL.VII/100), respectively.

Amendments made to the trial protocol before study completion included an adjustment in definition for one of the primary outcomes, prevalence of maternal anaemia at 36 weeks’ gestational age on July 27, 2021. We changed the definition of maternal anaemia to a Hb concentration of less than 11 g/dL instead of the initial less than 10 g/dL because we thought it necessary to identify all trial participants who were still anaemic irrespective of the severity. Additionally, we included prevalence of iron deficiency (serum ferritin <30 μg/L) and iron deficiency anaemia (Hb <11 g/dL and ferritin <30 μg/L) as secondary outcomes on July 20, 2023, because treatment with iron is likely to be more effective for iron deficiency than other causes of anaemia. Finally, we changed the determination of gestational age from ultrasound scans done in the first trimester, to scans done before 22 weeks’ gestation (July 27, 2021) because these scans were still acceptable as relatively accurate and most women did not have first trimester scans. This study was registered at the ISRCTN registry (ISRCTN63484804) on Dec 10, 2020, and ClinicalTrials.gov (NCT04976179) on April 7, 2021.

### Participants

Pregnant women aged 15–49 years and between 20 weeks’ and 32 weeks’ gestational age with Hb concentrations of less than 10 g/dL were invited to participate.^16^ Their Hb concentration was determined in the antenatal clinic from a fingerprick blood specimen as a point-of-care test using the Hemocue 301 device (HemoCue America, Brea, CA, USA). Gestational age was determined from the last menstrual period, or from an ultrasound scan done before 22 weeks’ gestation.

Exclusion criteria were substantial vaginal bleeding defined as any amount of bleeding from the vagina greater than spotting (light bleeding); blood transfusion or major surgery within the past 3 months; symptomatic anaemia; anaemia known to be unrelated to iron deficiency (eg, sickle cell anaemia diagnosed by haemoglobin electrophoresis, or HIV infection); clinically confirmed malabsorption syndrome; previous hypersensitivity to any form of iron; pre-existing maternal depression or other major psychiatric illness; immune-related diseases such as systemic lupus erythematosus or rheumatoid arthritis; or severe allergic reactions such as severe asthma. Written informed consent was obtained from all women older than 18 years before enrolment. Independent consent was obtained from those aged 15–17 years because of their pregnancy and Nigeria's consensus guidelines on adolescent participation in HIV and sexual and reproductive health research.^17^

### Randomisation and masking

Participants were randomly assigned to either receive intravenous iron (ferric carboxymaltose) or oral iron (ferrous sulphate) in a 1:1 ratio. Randomisation was done by study nurses or doctors using Sealed Envelope (London, UK), a web-based randomisation service, which also conceals allocation. Allocation was done using permuted balanced blocks of 4 × 4 stratified by study site. The study was primarily unmasked, because it would have been difficult to produce a placebo infusion resembling intravenous iron. Study nurses conducted participant follow-up; therefore, participants and site teams knew which group participants were randomly assigned to, but laboratory staff were masked. Apart from the data manager, no other study team members, including the trial biostatistician, had access to the unmasked data until the database was locked at study completion.

### Procedures

Enrolled women were randomly assigned to either receive a single infusion of ferric carboxymaltose (20 mg/kg to a maximum total dose of 1000 mg in 200 mL of 0·9% normal saline as an infusion over 15–20 min; intervention group) or oral ferrous sulphate (200 mg tablets containing 65 mg elemental iron three times daily until 6 weeks after delivery; control group). Ferrous sulphate was given three times daily per standard practice for the treatment of anaemia in pregnancy in Nigeria.^18^ All participants received 5 mg of folic acid once daily and 100 mg of vitamin C three times daily until the end of pregnancy. Additionally, monthly sulphadoxine–pyrimethamine and insecticide-treated bednets were provided for malaria prevention.

At enrolment, research nurses administered the English version of the Edinburgh Postnatal Depression Scale (EPDS) tool for depression screening to all participants.^19^ Additionally, participants had blood collected for complete blood count, serum ferritin and serum phosphate assessments, and point-of-care testing for malaria parasitaemia; those who screened positive were treated with artemisinin-based combination therapy. Participant pregnancies were managed as per routine, with an appointment every 4 weeks up to 28 weeks’ gestational age, an appointment every 2 weeks up to 36 weeks’ gestational age, and weekly until delivery. After delivery, mothers and infants were followed up at 2 weeks and 6 weeks postpartum.

Follow-up laboratory assays included complete blood count, serum iron, total iron binding capacity and transferrin saturation, serum ferritin concentration, and maternal serum phosphate concentration assessed at specific time intervals (appendix 1 p 4). Cord blood phosphate was assayed at delivery. Trial participants were further assessed for depression using the EPDS tool at 36 weeks’ gestation. All trial participants who screened positive for depression (EPDS score ≥10) were referred to the trial psychiatrists for further evaluation. Neonatal outcomes such as infant birthweight, head circumference, and length were recorded at delivery. Neonatal survival was recorded at delivery and at 6 weeks postpartum.

Data on adverse drug events were collected at each visit with a study-designed adverse event reporting form. The data included information on severity, frequency, relationship to trial drug, actions taken, and outcome of the event. Maternal vital signs were recorded at each visit. For those on oral iron, drug adherence was calculated as the mean of all individual adherence, estimated as the percentage of the sum total of all pills dispensed to the participant during the study that were consumed. The schedule for trial participant assessments is presented in appendix 1 (p 4). SMS appointment reminders were sent to all participants to minimise loss to follow-up. Participants who missed a visit were promptly contacted by telephone to establish reasons for the missed visit and for rescheduling. When a trial participant could not be reached, she was visited at home for data and blood specimen collection. A record log of all protocol deviations was kept. Data were collected and managed on the Research Electronic Data Capture platform.

### Outcomes

Primary outcome measures were prevalence of maternal anaemia (Hb <11 g/dL) at 36 weeks’ gestation, and preterm birth (before 37 weeks’ gestation). Secondary outcome measures (maternal) were: prevalence of iron deficiency (serum ferritin <30 μg/L) and iron deficiency anaemia at 36 weeks’ gestation; depression at 36 weeks’ gestation and at 2 weeks postpartum; Hb concentration at 4 weeks after treatment initiation and at delivery; safety of iron, including incidence of hypophosphataemia; and severity of adverse events at day 1 and 4 weeks after enrolment, at 36 weeks’ gestational age, and at 6 weeks postpartum; prevalence of severe adverse events, specifically postpartum haemorrhage (blood loss exceeding 500 mL for vaginal delivery and 1000 mL for caesarean section), sepsis, shock, and need for blood transfusion. Secondary outcome measures for infants were incidence of small for gestational age (defined as birthweight below the 10th percentile for gestational age),^20^ incidence of low infant birthweight (<2·5 kg), stillbirth or neonatal mortality (within 28 days of life), cord blood hypophosphataemia, proportion of infants breastfed at age 2 weeks and 6 weeks, and proportion receiving up-to-date vaccination (ie, receiving BCG, oral polio, and hepatitis vaccines) by age 6 weeks (appendix 1 p 4).

Outcomes for twins and triplets were analysed as a single outcome. For birthweight, the mean birthweight for the set was analysed. For stillbirth, neonatal death, and vaccination status, the worst outcome was analysed.

Maternal adverse events considered were fever, weakness, chills or rigors, headache, myalgia, dizziness, gastrointestinal symptoms, palpitations, insomnia, pruritus, cough, tinnitus, abnormal gait, tremor, clonus, or dyskinesia. Hypophosphataemia was also considered along with severe adverse events (those requiring hospital admission, or maternal or perinatal death).

### Statistical analysis

We estimated a sample size of 1056 to detect a difference of 14% (16% intravenous *vs* 30% oral) in the prevalence of anaemia at 36 weeks’ gestation. Assumptions were based on a multicountry study involving Europe, Asia, and Australia, at 90% power, adjusting for 15% attrition.^21^ The estimated sample size was sufficient to detect a difference of 6% in the incidence of preterm births. Based on data from Nigerian studies, we estimated the incidence of preterm births at approximately 25%.^22^, ^23^, ^24^ Two-tailed test of hypothesis based on the Z test for proportions and 95% CI were assumed. Sample size estimation did not consider subgroup analyses. A statistical analysis plan was established before datalock (appendix 1 pp 7–26).

The main analyses were by intention to treat and included all participants with available outcome data according to the treatment they were assigned to at randomisation. Continuous outcomes were summarised using the n, mean, and SD, while categorical outcomes were summarised using the n, frequency, and percentage of observed levels. Log-binomial regression models were used to assess the treatment effect on dichotomous categorical outcomes, and risk ratios (RRs) were presented with 95% CI calculated from the variance of the beta coefficients.

In predefined subgroup analyses, log-binomial models were conducted stratified by state (Lagos *vs* Kano) and facility type (primary *vs* secondary *vs* tertiary) to evaluate the extent to which the treatment effect on primary outcomes varied by these contextual factors, and p values were obtained from likelihood ratio tests. Participants who had anaemia, but not iron deficiency anaemia, were described as having non-iron deficiency anaemia. In a subgroup analysis, the extent to which the treatment effect differed in subgroups of women with iron deficiency versus non-iron deficiency anaemia at enrolment was evaluated using likelihood ratio tests.

The 4-week increase in maternal Hb concentration from treatment initiation was presented in graphs as line charts, and p values were obtained from a random-intercepts linear mixed effects regression model and an interaction term for the timepoint (0 *vs* 4 weeks) and the treatment group (intravenous *vs* oral iron). The analysis was repeated in subgroups of women with iron deficiency versus non-iron deficiency anaemia at enrolment. Log-binomial regression models were used to evaluate the treatment effect on the occurrence of hypophosphataemia at each timepoint.

All the preceding analyses were prespecified in the statistical analysis plan. In a post hoc analysis, we compared the baseline characteristics in Kano and Lagos, and obtained p values from χ^2^, Fisher's exact, Wilcoxon rank-sum, and *t* tests, as appropriate. We also conducted a subgroup analysis by gestational age at trial enrolment (<28 weeks *vs* ≥28 weeks).

Complete cases were analysed, and there was no imputation for missing data. Statistical analysis was done with RStudio 1.0.153. An independent statistician reviewed the codes. A data and safety monitoring committee was established to monitor trial data authenticity and safety.

### Role of the funding source

The funder had no role in the study design, data collection, data analysis, data interpretation, or writing of the report.

## Results

**Figure 1:**
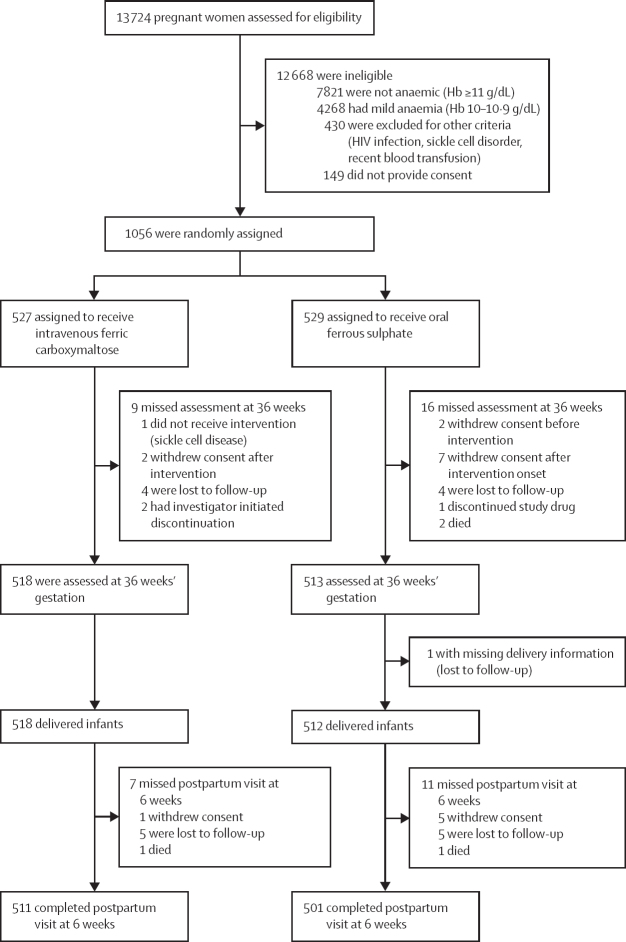
Trial profile Hb=haemoglobin.

We screened 13 724 pregnant women between Aug 10, 2021, and Dec 15, 2022. Of these, we excluded 7821 who were not anaemic, 4268 who had mild anaemia, 430 for other reasons (such as HIV infection or sickle cell disorder), and 149 who did not provide consent. 1056 consented to participate and were randomly assigned to either the intravenous or oral administration groups. 527 were assigned to the intravenous ferric carboxymaltose group and 529 were assigned to the oral ferrous sulphate group. 518 in the intravenous group were assessed at 36 weeks’ gestational age and after 518 deliveries, and 511 completed the 6 weeks postpartum visit. 513 in the oral ferrous sulphate group were assessed at 36 weeks’ gestational age and after 512 deliveries, and 501 completed the 6 weeks postpartum visit (figure 1). The last 6-week follow-up visit was on June 15, 2023. The mean drug adherence rate for those who had oral iron was 94·4% (SD 11·8).

The baseline characteristics were well balanced by study group (table 1). The prevalence of iron deficiency was 39·8% (420 of 1056) based on ferritin less than 30 μg/L. There was no significant difference in the prevalence of maternal anaemia at 36 weeks’ gestation in the intravenous versus oral iron groups (299 [58%] of 517 *vs* 305 [61%] of 503; RR 0·95, 95% CI 0·85–1·06; p=0·36) or in preterm birth (73 [14%] of 518 *vs* 77 [15%] of 513; 0·94, 0·70–1·26; p=0·66; table 2).

**Table 1:** Baseline characteristics

		**Intravenous iron group (n=527)**	**Oral iron group (n=529)**
Age, years		28 (23–33)	28 (24–33)
First pregnancy*		192 (36%)	187 (35%)
Gestational age at enrolment, weeks		25·0 (22–28)	25·0 (22–28)
	<20	5 (1%)	0
	20–27	351 (67%)	381 (72%)
	28–42	171 (32%)	148 (28%)
Educational attainment			
	No formal education	43 (8%)	26 (5%)
	Primary or secondary	322 (61%)	347 (66%)
	Tertiary or above	161 (31%)	154 (29%)
	Missing	1 (<1%)	2 (<1%)
Not married		27 (5%)	19 (4%)
Urban place of residence		480 (91%)	483 (91%)
State			
	Kano	267 (51%)	270 (51%)
	Lagos	260 (49%)	259 (49%)
Ethnicity			
	Hausa	259 (49%)	257 (49%)
	Igbo	59 (11%)	74 (14%)
	Yoruba	177 (34%)	157 (30%)
	Others	32 (6%)	39 (7%)
	Missing	0	2 (<1%)
Hb concentration, g/dL		9·14 (0·70)	9·15 (0·69)
Severe anaemia (Hb <7 g/dL)		11 (2%)	8 (2%)
Ferritin concentration, μg/L†		66·4 (108·1)	77·1 (107·8)
Iron deficiency (ferritin <30 μg/L)			
	No	303 (58%)	326 (62%)
	Yes	220 (42%)	200 (38%)
	Missing	4 (1%)	3 (1%)

Data are n (%), median (IQR), or mean (SD). Hb=haemoglobin.

* There were four missing observations for parity (one in the intravenous group and three in the oral group).

† There were seven missing observations for ferritin (four in the intravenous group and three in the oral group).

**Table 2:** Treatment effect on primary and secondary outcomes

		**Intravenous iron group (n=527)**	**Oral iron group (n=529)**	**Risk ratio (95% CI)**	**p value**
**Primary outcome**					
Maternal anaemia (Hb <11 g/dL) at 36 weeks' gestation*		299/517 (58%)	305/503 (61%)	0·95 (0·85–1·06)	0·36
Preterm birth		73/518 (14)	77/513 (15%)	0·94 (0·70–1·26)	0·66
**Secondary outcomes**					
Moderate or severe anaemia (Hb <10 g/dL)*		115/503 (23%)	104/517 (20%)	0·88 (0·70–1·11)	0·29
Iron deficiency at 36 weeks' gestation*		23/516 (5%)	82/500 (16%)	0·27 (0·17–0·42)	<0·0001
	Iron deficiency anaemia at 36 weeks' gestation*	11/516 (2%)	48/498 (10%)	0·22 (0·12–0·42)	<0·0001
Moderate or severe iron deficiency anaemia		5/516 (1%)	19/498 (4%)	0·25 (0·10–0·67)	0·0058
Maternal depression†					
	Prevalence at 36 weeks' gestation	25/444 (6%)	19/427 (4%)	1·28 (0·72–2·29)	0·40
	Proportion achieving minimal important change in EPDS	60/444 (14%)	60/426 (14%)	0·97 (0·70–1·36)	0·87
Postpartum haemorrhage		7 (1%)	5 (1%)	1·41 (0·45–4·72)	0·56
Blood transfusion		3 (1%)	4 (1%)	0·75 (0·15–3·42)	0·71
Low birthweight (<2·5 kg)‡		36/406 (9%)	31/396 (8%)	1·13 (0·72–1·80)	0·60
Small for gestational age (<10th percentile for gestational age)‡		83/406 (20%)	65/395 (17%)	1·25 (0·93–1·68)	0·14
Stillbirth		17/519 (3%)	15/512 (3%)	1·12 (0·56–2·25)	0·75
Neonatal death‡		5/468 (1%)	9/473 (2%)	1·00 (0·88–1·14)	>0·99
Breastfeeding at 2 weeks postpartum§		423/455 (93%)	403/458 (88%)	1·06 (0·92–1·21)	0·43
Breastfeeding at 6 weeks postpartum§		407/456 (89%)	397/457 (87%)	1·03 (0·89–1·18)	0·70
Vaccination up to date by 6 weeks postpartum§		390/444 (88%)	388/436 (89%)	0·98 (0·85–1·12)	0·72

Data are n/N (%). EPDS=Edinburgh Postnatal Depression Scale. Hb=haemoglobin concentration.

* Of the 1031 participants assessed at 36 weeks (518 in the intravenous group and 513 in the oral group), there were missing observations in the analyses for anaemia (one in the intravenous group and ten in the oral group), iron deficiency (two in the intravenous group and 13 in the oral group), and iron deficiency anaemia (two in the intravenous group and 15 in the oral group).

† Only participants with term deliveries could have a suitable EPDS measure for depression analysis (881 had a term delivery but 871 had an EPDS measure for depression analysis [444 in the intravenous group and 427 in the oral group]; nine in the oral group had a term delivery but missed the EPDS measure). Of these, there were missing observations for 74 participants in the intravenous group and 86 participants in the oral group.

‡ Of the 1030 participants with delivery information (518 in the intravenous group and 512 in the oral group), there were missing observations in the analyses for birthweight (112 in intravenous group and 116 in the oral group), small for gestational age (112 in the intravenous group and 117 in the oral group), and neonatal death (50 in the intravenous group and 39 in the oral group).

§ Of the 1012 participants who completed postpartum visits (511 in the intravenous group and 501 in the oral group), there were missing observations in the analyses for breastfeeding at 2 weeks (56 in the intravenous group and 43 in the oral group), breastfeeding at 6 weeks (55 in the intravenous group and 44 in the oral group), and vaccinations (67 in the intravenous group and 65 in the oral group).

**Figure 2:**
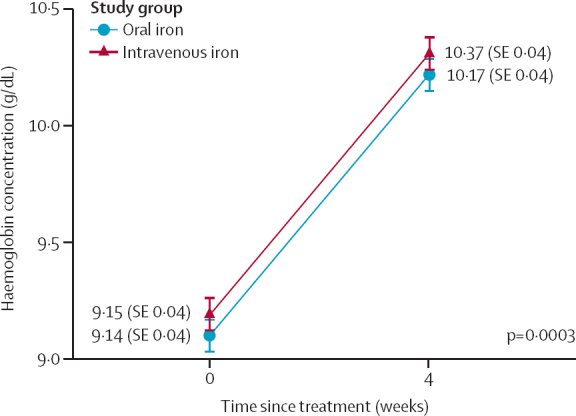
Treatment effect on the increase in maternal haemoglobin concentration Data are presented as mean (95% CI). The p value was calculated from a likelihood ratio test comparing the model with the interaction term to the model without. The χ^2^ was 20·5 (df 4).

The slope of the change in the mean Hb concentration between baseline and 4 weeks significantly differed in the intravenous and oral group (p_interaction_=0·0003, figure 2). Intravenous iron was also more effective at reducing iron deficiency (RR 0·27, 95% CI 0·17–0·42; p<0·0001) and iron deficiency anaemia (0·22, 0·12–0·42; p<0·0001) at 36 weeks (table 2). There was no significant difference in both groups in the prevalence of depression at any timepoint nor in the prevalence of moderate to severe anaemia at 36 weeks’ gestation.

Intravenous iron led to a higher mean Hb concentration from baseline to 4 weeks in both the iron-deficient (p=0·023) and non-iron deficient subgroups (p=0·0078). However, the slope of the treatment effect in both subgroups differed significantly (p_interaction_<0·0001; appendix 1 p 6).

In the subgroup analysis by baseline iron deficiency status at enrolment, intravenous iron was significantly more effective than was oral iron in reducing the prevalence of maternal anaemia (Hb <11 g/dL) at 36 weeks’ gestation in the iron deficiency anaemia versus non-iron deficiency anaemia subgroup (p_interaction_=0·039; table 3). Although intravenous iron reduced the prevalence of maternal anaemia compared with oral iron in the iron deficiency subgroup (RR 0·83, 95% CI 0·71–0·98), it had no effect in the non-iron deficiency subgroup (1·04, 0·91–1·18). The effect of intravenous versus oral iron on preterm birth did not vary by presence of iron deficiency at enrolment. At 36 weeks’ gestation, iron deficiency was corrected in 216 (98%) of 220 women with baseline iron deficiency in the intravenous group, but only in 173 (91%) of 191 women with baseline iron deficiency in the oral group.

**Table 3:** Subgroup analyses of primary outcomes

		**Intravenous iron group (n=527)**	**Oral iron group (n=529)**	**Risk ratio (95% CI)**	**p value for interaction**
**Maternal anaemia (Hb <11 g/dL) at 36 weeks' gestation**					
Baseline iron deficiency status					
	Iron deficient	119/220 (54%)	124/191 (65%)	0·83 (0·71–0·98)	0·040
	Non-iron deficient	178/295 (60%)	181/309 (59%)	1·04 (0·91–1·18)	..
Gestational age at enrollment, weeks					
	<28	199/348 (57%)	199/360 (55%)	1·03 (0·91–1·18)	0·61
	≥28	100/169 (59%)	106/143 (74%)	0·80 (0·68–0·93)	..
State (regional) differences					
	Lagos	145/252 (58%)	152/244 (62%)	0·93 (0·80–1·07)	0·60
	Kano	154/265 (58%)	153/257 (60%)	0·98 (0·85–1·13)	..
Facility differences					
	Primary	80/122 (66%)	80/124 (65%)	1·02 (0·85–1·23)	0·50
	Secondary	197/353 (56%)	207/399 (52%)	0·91 (0·81–1·04)	..
	Tertiary	22/42 (52%)	18/38 (47%)	1·11 (0·71–1·76)	..
**Preterm delivery**					
Baseline iron deficiency status					
	Iron deficient	33/220 (15%)	33/195 (17%)	0·89 (0·57–1·38)	0·77
	Non-iron deficient	40/297 (13%)	44/316 (14%)	0·97 (0·65–1·44)	..
Gestational age at enrollment, weeks					
	<28	45/350 (13%)	60/367 (16%)	0·79 (0·55–1·12)	0·010
	≥28	28/169 (17%)	17/145 (12%)	1·41 (0·82–2·53)	..
State (regional) differences					
	Lagos	25/254 (10%)	40/251 (16%)	0·62 (0·38–0·98)	0·018
	Kano	48/265 (18%)	37/261 (14%)	1·28 (0·86–1·91)	..
Facility differences					
	Primary	21/123 (17%)	16/126 (13%)	1·34 (0·74–2·50)	0·39
	Secondary	49/354 (14%)	58/348 (17%)	0·83 (0·58–1·18)	..
	Tertiary	3/42 (7%)	3/38 (8%)	0·90 (0·18–4·64)	..

Data are n/N (%), unless otherwise specified. p values for interaction are based on a likelihood ratio test comparing a model with an interaction term for each treatment baseline iron deficiency status to model without the interaction term. Hb=haemoglobin. RR=risk ratio

In subgroup analyses by gestational age at enrolment, there were no significant differences in the RRs for maternal anaemia (p_interaction_=0·61), but the RRs for preterm birth significantly varied (p_interaction_=0·0087). The RR of preterm birth was 0·79 (95% CI 0·55–1·12) among those enrolled before 28 weeks’ gestational age and 1·41 (0·82–2·53) among those enrolled at a gestational age of 28 weeks and above.

With respect to differences between the two states—Kano and Lagos—of participants whose Hb concentration was reported, we did not find a significant difference in the prevalence of maternal anaemia at 36 weeks’ gestation between those who received intravenous iron (154 [58%] of 265 women for Kano and 145 [58%] of 252 women for Lagos) and those who received oral iron (153 [59%] of 258 women for Kano and 152 [62·0%] of 245 women for Lagos; p_interaction_=0·60). However, on assessment of preterm delivery between the two states, there was a reduction in the incidence of preterm delivery for those in the intravenous group in Lagos State. The incidence of preterm delivery in Lagos State was 25 (10%) of 254 women in the intravenous group versus 40 (16%) of 251 women in the oral group (RR 0·62 [95% CI 0·38–0·98]; p_interaction_=0·018). There was no significant difference in the incidence of preterm delivery by treatment group in Kano State (table 3). To contextualise these findings, we compared the baseline characteristics in Kano and Lagos, and found meaningful differences (appendix 1 p 5). Participants in Kano had completed fewer educational milestones, were less likely to be in their first pregnancy, more homogenously Hausa in their ethnicity, and more likely to have delivered at home by unskilled attendants or by a traditional birth attendant.

We recorded four maternal deaths, all unrelated to study drugs: two in the intravenous group and two in the oral group. Causes of death were antepartum haemorrhage from placenta praevia (n=1) and placenta abruption (n=1), both in the control group, and from puerperal sepsis (n=1) and postpartum haemorrhage (n=1), both in the intravenous group.

There were three serious adverse events (grade 3): diarrhoea, hypertension, and postpartum haemorrhage, and only one (diarrhoea) was possibly related to the study drug (ferrous sulphate). All serious adverse events resolved following treatment and care.

We recorded 24 grade 1 and 2 adverse events affecting 21 participants. Of these, nine adverse events affected nine people in the intravenous group, and 15 adverse events affected 12 people in the oral group. In the oral group, diarrhoea occurred in six participants and vomiting occurred in three participants. The other 15 adverse events were in less than three participants each and did not differ by study group: nausea (n=3), fatigue (n=3), headache (n=3), hypertension (n=2), congenital familial and genetic disorders (n=1), chills (n=1), cough (n=1), and postpartum haemorrhage (n=1). None of the participants reported constipation.

23 (4%) of 526 participants in the intravenous group had transient hypotension during or following intravenous iron administration. None of these cases were symptomatic. Blood pressure was not measured after administration in the oral group.

The incidence of hypophosphataemia at the 4-week follow-up visit was considerably higher in the intravenous group (53 [11%] of 498) than in the oral group (five [1%] of 477). However, the occurrence of hypophosphatemia did not significantly differ by treatment group at any other timepoint (appendix 1 p 5).

## Discussion

In this randomised controlled trial of 1056 pregnant women in Nigeria, we found no evidence that intravenous iron (ferrous carboxymaltose) was superior to oral iron (ferrous sulphate) to treat anaemia or prevent adverse clinical maternal or fetal outcomes. However, intravenous iron significantly reduced the prevalence of iron deficiency and iron deficiency anaemia. Intravenous iron also led to a higher increase in mean Hb concentration at 4 weeks after enrolment and was administered safely without any serious drug-related adverse events. Furthermore, in the subgroup of pregnant women with iron deficiency anaemia at enrolment, intravenous iron more effectively reduced maternal anaemia compared with oral iron.

Our findings are in contrast to those of a multicentre international trial, which found that ferric carboxymaltose caused a significantly greater reduction in the prevalence of anaemia among pregnant women with iron deficiency anaemia in five high-income and two upper-middle-income countries.^25^ However, the women enrolled in that study were those with iron deficiency anaemia ab initio**,** whereas ours was conducted in a lower-middle-income country where ferritin measurements for iron deficiency anaemia screening are not standard of care, due to cost and turnaround time. Another trial conducted in Malawi had results similar to ours: ferric carboxymaltose was not better than standard oral iron treatment in reducing anaemia at 36 weeks’ gestation.^15^ As in our trial, the Malawi trial found intravenous iron more effective in reducing the prevalence of iron deficiency anaemia.

Trials of other intravenous iron formulations have also reported improvements in Hb and ferritin concentrations, and a recent meta-analysis found that the differential effect was greater in studies that enrolled pregnant women with Hb concentrations of less than 9 g/dL.^13^, ^26^ Available evidence therefore indicates that intravenous iron treatment is more effective than oral iron to replenish iron stores and thereby improve Hb concentration, especially if patients were correctly identified as iron deficient, or had iron deficiency anaemia.

At 36 weeks’ gestation, a substantial proportion of women in both the intravenous (58%) and oral groups (61%) had persistent anaemia (Hb <11 g/dL) but only 23% and 20%, respectively, had moderate or severe anaemia (Hb <10 g/dL). The higher prevalence of mild anaemia might reflect the physiologically normal plasma volume expansion in pregnancy.^27^

Our null findings concerning the primary outcome of preterm birth might be potentially related to the limited statistical power. The sample size estimation assumed a 25% rate of preterm births,^22^, ^23^, ^24^ but this rate was only 14·5% in our study, thus the study was likely underpowered to measure this outcome. Furthermore, we might not have detected a difference in effect because most participants were enrolled well after 20 weeks’ gestation and, thus, we were unable to explore the effect of duration of treatment in subgroup analysis. However, our findings are consistent with those of other randomised controlled trials of intravenous versus oral iron.^13^

We did not find any significant differences in the effect of intravenous versus oral iron on non-haematological clinical outcomes. We examined the risk of postpartum depression as an outcome when comparing intravenous versus oral iron but did not find any significant differences despite our relatively large sample size. Furthermore, randomised trials of oral supplementation versus placebo have not found consistent effects on maternal and fetal outcomes.^28^, ^29^

Our finding of an effect of intravenous iron on preterm birth in Lagos but not in Kano State, and not overall, might be partially explained by the lower proportion of women with formal education and the higher proportion of home delivery among our Kano participants. It is probable that these characteristics play a larger role in the incidence of preterm birth in Kano as opposed to anaemia. Low level of education and low antenatal clinic attendance have been associated with a higher risk for preterm births in Nepal.^30^ Low antenatal care also increased the odds of preterm births in a Nigerian study.^31^

There were no marked differences in safety for ferric carboxymaltose and oral iron in this study. Other studies have found no difference or a lower incidence of adverse events with ferric carboxymaltose compared with oral iron.^15^, ^32^ Similar to the two previous trials with ferric carboxymaltose in pregnant women,^15^, ^25^ we found a higher incidence of transient hypophosphataemia in the intravenous group compared with the oral ferrous sulphate group, although this did not occur at any other timepoint after 4 weeks post-treatment. Our observation of no difference in the incidence of hypophosphataemia in cord blood is both original and reassuring and suggests a lack of persistent hypophosphataemia in the neonate, even if there had been an initial reduction in phosphate concentration. This finding affirms the safety of ferric carboxymaltose in the unborn fetus, an important consideration for any drug administered during pregnancy. Unlike in the study by Breymann and colleagues,^25^ we did not observe any hypersensitivity reactions. The finding of transient but non-clinically relevant mild hypotension in 4% of participants in the intravenous iron group is worthy of note to ensure monitoring for at least 30 min before discharge, as practised in our trial.

To our knowledge, this is the largest randomised trial evaluating the effectiveness and safety of ferric carboxymaltose for treating iron deficiency anaemia in pregnancy, and the first in west Africa. We evaluated a large number of clinical outcomes and tested for hypophosphataemia, a known side-effect of ferric carboxymaltose, in cord blood. Additionally, compliance with treatment allocation and follow-up rates were high and a significant effect on iron deficiency anaemia was reported. However, there are some limitations. Even a trial of this size is not large enough to rule out small but potentially clinically important effects such as preterm birth and postpartum haemorrhage. In our study setting, the interpretation of what constitutes postpartum haemorrhage is limited by the use of visual assessment to determine blood loss at delivery, a method which is generally subjective.^33^ There is also a risk for bias in EPDS scoring, because the assessors were unmasked. The participants in the oral group had 195 mg elemental iron daily, which is higher than the WHO recommended 120 mg daily dose for treatment of iron deficiency. However, as the adherence rate was much higher than the 33–66% previously reported in Nigeria,^8^ this suggests that the effect of ferric carboxymaltose might be higher in routine practice. Pregnant women with mild anaemia and women living with HIV who might have benefited from the study were excluded, which might affect the generalisability of our findings.

Although our study did not find a significant difference in anaemia prevalence near term, we were able to establish that a single dose of 1000 mg ferric carboxymaltose given during pregnancy is more effective than oral ferrous sulphate in treating iron deficiency and iron deficiency anaemia and is safe. As women in the iron deficiency anaemia subgroup had a significant reduction of anaemia, there is difficulty with diagnosing iron deficiency in our setting due to the cost of ferritin, and as ferric carboxymaltose is safe, we recommend that it should be considered in anaemic women and prioritised in women with confirmed iron deficiency. Future studies should concentrate on assessing the clinical effectiveness of intravenous iron in severe anaemia in pregnancy (Hb <7g/dL). The potential effect of intravenous iron on preterm birth should also be re-examined, with analyses considering related socioeconomic factors.

### IVON Trial Investigators

### Contributors

### Equitable partnership declaration

### Data sharing

Data will be deposited in the Open Science Framework. Pending data deposition in the repository, data are available on reasonable request from the Principal Investigator of the IVON trial who is the lead and corresponding author of this manuscript.

For more on the **ALERT project** see https://alert.ki.se/

## Declaration of interests

KSA reports participation on the ALERT project Data Safety Monitoring Board. All other authors declare no competing interests.

## Supplementary Materials

Supplementary appendix 1

Equitable Partnership Declaration
