# Supplementary material for: Intravenous versus oral iron for anaemia among pregnant women in Nigeria (IVON): an open-label, randomised controlled trial
Source: Lancet Glob Health. 2024 Sep 18;12(10):e1649–59. doi: 10.1016/S2214-109X(24)00239-0 (PMC11420468; doi:10.1016/S2214-109X(24)00239-0)
Supplement: Supplementary appendix 1 [file mmc1.pdf]

THE LANCET
Global Health

Supplementary appendix 1

This appendix formed part of the original submission and has been peer reviewed. We post it as supplied by the authors.

Supplement to: Afolabi BB, Babah OA, Adeyemo TA, et al. Intravenous versus oral iron for anaemia among pregnant women in Nigeria (IVON): an open-label, randomised controlled trial. *Lancet Glob Health* 2024; **12:** e1649–59.

Appendix

Table SM1

Table SM1. Study sites for the IVON trial

State	Level of healthcare	Study site
Lagos	Primary	Simpson Primary Health Centre, Ebute-Metta, Lagos
	Primary	Iwaya Primary Health Centre, Yaba, Lagos
	Secondary	Mother and Child Centre, Amuwo-Odofin, Lagos
	Secondary	Lagos Island Maternity Hospital, Lagos
	Tertiary	Lagos University Teaching Hospital, Idi-Araba, Lagos
Kano	Primary	Kabuga Primary Health Centre, kano
	Primary	Sharada Primary Health Centre, Kano
	Primary	Kumbotsu Comprehensive Health Centre, Kumbotsu, Kano
	Secondary	Nuhu Bammali General Hospital, Kano
	Secondary	Sheikh Muhammad Jiddah General Hospital, Kano
	Tertiary	Aminu Kano Teaching Hospital, Kano

Table SM2

Table SM2. Rationale for defining inclusion criteria and cut-off values for participants' enrolment and outcome measures in the IVON TRIAL

Variable selection criteria or cut-off value	Rationale for criteria/cut-off applied
Haemoglobin concentration <10g/dL to qualify for inclusion into the study.	We included pregnant women with Hb <10g/dL (that is, moderate or severe anaemia only) because most of the morbidities and mortalities that are associated with anaemia occur at these Hb levels. Secondly, considering the haematological changes in pregnancy in terms of physiologic haemodilution, it will be easier to observe improvements when the starting Hb level is lower. Furthermore, we felt it would be difficult to advocate for the ethical use of intravenous iron to treat mild anaemia, especially in a setting where intravenous iron is not frequently used for anaemia treatment.
Malaria as a known risk factor for anaemia in pregnancy. The situation in Nigeria and this clinical trial.	We ensured all participants were screened and treated for malaria with an artemisinin-based combination therapy at enrolment into this study. We also ensured they all had regular prophylaxis afterwards with intermittent preventive therapy for malaria using Sulphadoxine-Pyrimethamine. The reason for this is that malaria is endemic in Nigeria with prevalence varying between states. We found a prevalence of 5.9% for malaria among pregnant women with moderate or severe anaemia in a sub-study from this clinical trial which examined the prevalence and risk factors for iron deficiency anaemia during pregnancy;[1] this was not likely to have played a large role in anaemia in this setting.
Human immunodeficiency virus infection (HIV) as an exclusion criterion.	We excluded women who have HIV infection because of its association with anaemia. The pooled prevalence of HIV in pregnancy in Nigeria is 7.2%.[2] It is reported that some of the anti-retroviral drugs may have effects on haematopoiesis.[3,4] Furthermore, opportunistic infections that may occur in HIV patients are often associated with inflammation which can affect the diagnosis of iron deficiency with serum ferritin.
Haemoglobinopathies like sickle cell disorder as an exclusion criterion.	We excluded pregnant women with sickle cell disease as iron is not routinely prescribed in them during pregnancy in our setting as they are often iron replete from frequent blood transfusions and recurrent haemolysis.
Haemoglobin concentration <11g/dL for outcome (anaemia prevalence) assessment.	Based on WHO definition for anaemia in pregnancy, we used Hb < 11g/dl for the outcome measurement , since the goal of anaemia treatment is irrespective of the severity. We were interested in knowing if complete treatment was achieved, and not merely if the severity of anaemia was reduced.
Serum ferritin <30ng/ml defining iron deficiency	Though WHO recommended serum ferritin level of 15µg/L as threshold for diagnosis of iron deficiency during pregnancy, we used a higher cut off of 30µg/L to minimize the impact of probable inflammation because we did not assess inflammation using biomarkers like C-reactive protein in this study.[5] In addition, using a cutoff of 30 ug/L has been shown to improve the sensitivity from 25 to 92 percent, and specificity remains high at 98 percent.[6] In Van de Broek's study

	based on bone marrow aspirates of pregnant women in Malawi,[7] the serum ferritin cut-off of 30 µg/L was found to be the best with a sensitivity of 90% and a specificity of 85% for the detection of iron deficiency.
Hypothesis for inclusion of vaccine uptake as a secondary outcome measure.	Considering that our women often take their babies for immunization themselves, we hypothesized that their ability to do this is an indication of maternal wellness and an indirect assessment of their quality of life which can be affected by anaemia.

1. *Babah OA, Akinajo OR, Beňová L, Hanson C, Abioye AI, Adaramoye VO, Adeyemo TA, Balogun MR, Banke-Thomas A, Galadanci HS, Sam-Agudu NA, Afolabi BB, Larsson EC. Prevalence of and risk factors for iron deficiency among pregnant women with moderate or severe anaemia in Nigeria: a cross-sectional study. BMC Pregnancy Childbirth. 2024 Jan 5;24(1):39.*
2. *Ozim CO, Mahendran R, Amalan M, Puthussery S. Prevalence of human immunodeficiency virus (HIV) among pregnant women in Nigeria: a systematic review and meta-analysis. BMJ Open [Internet]. 2023 2023/03//; 13(3):[e050164 p.]. Available from: https://bmjopen.bmj.com/content/bmjopen/13/3/e050164.full.pdf*
3. *Berhane Y, Haile D, Tolessa T. Anemia in HIV/AIDS Patients on Antiretroviral Treatment at Ayder Specialized Hospital, Mekele, Ethiopia: A Case-Control Study. J Blood Med. 2020 Oct 21;11:379-387.*
4. *Volberding PA, Levine AM, Dieterich D, Mildvan D, Mitsuyasu R, Saag M, et al. Anemia in HIV Infection: Clinical Impact and Evidence-Based Management Strategies. Clinical Infectious Diseases. 2004;38(10):1454-63.*
5. *WHO. WHO guideline on use of ferritin concentrations to assess iron status in individuals and populations. Apr 2020. Available at: https://www.who.int/publications/i/item/9789240000124*
6. *Mast AE, Blinder MA, Gronowski AM, Chumley C, Scott MG. Clinical utility of the soluble transferrin receptor and comparison with serum ferritin in several populations. Clin Chem. 1998 Jan;44(1):45-51. PMID: 9550557.*
7. *van den Broek NR, Letsky EA. Etiology of anemia in pregnancy in south Malawi. Am J Clin Nutr. 2000 Jul;72(1 Suppl):247S-256S. doi: 10.1093/ajcn/72.1.247S. PMID: 10871590*

Table SM3

Table SM3. Schedule of assessments

Visit	Treatment (Baseline)	4 weeks post-enrolment	36 weeks gestational age	Delivery	2 weeks postpartum	6 weeks postpartum
Socio-demographics	X					
Physical exam	X	X	X	X	X	X
Haemoglobin	X	X	X	X	X	X
Malaria	X					
Full blood count	X + 4 weeks after	X	X	X		X
Iron panel	X + 4 weeks after	X	X	X		X
Maternal serum phosphate	X + 4 weeks after	X	X	X		X
EPDS	X	X	X		X	
Adverse events	X	X	X	X	X	X
Child immunization status						X

Table SM4

Table SM3. Differences in participants' characteristics by state

	Kano (N=537)	Lagos (N=519)	P-value
**Age (years),** Mean (SD)	26.8 (5.98)	29.9 (5.65)	0.53
**First pregnancy**	147 (27.4%)	232 (44.7%)	<0.001
**Gestational age at enrollment (weeks),** Median (IQR)	26 (23, 28)	24 (22, 28)	0.29
**Educational attainment**			
No formal education	64 (11.9%)	5 (1.0%)	<0.001
Primary or secondary	419 (78.0%)	250 (48.2%)	
Tertiary	54 (10.1%)	261 (50.3%)	
**Haemoglobin concentration at enrolment (g/dL),** Mean (SD)	9.02 (0.79)	9.28 (0.55)	0.39
**Unmarried**	2 (0.4%)	44 (8.5%)	<0.001
**Urban place of residence**	503 (93.7%)	460 (88.6%)	0.005
**Ethnicity**			
Hausa	511 (95.2%)	5 (1.0%)	<0.001
Igbo	0 (0%)	133 (25.6%)	
Yoruba	1 (0.2%)	333 (64.2%)	
Others	25 (4.7%)	46 (8.9%)	
**Severe anaemia (Hb <7g/dL)**	16 (3.0%)	3 (0.6%)	0.007
**Delivery at home/TBA**	206 (38.4%)	41 (7.9%)	<0.001

Table SM5

Table SM4. Incidence of hypophosphataemia during by treatment arm (N=1,056)

Timing	IV	Oral	RR	95% CI	*P-value*
At 4 weeks post-enrolment visit	53/498 (10.6)	5/477 (1.0)	10.2	(4.53, 29.0)	<0.001
At delivery	4/418 (1.0)	7/442 (1.6)	0.60	(0.16, 1.99)	0.42
At 6 weeks postpartum visit	3/415 (0.7)	1/430 (0.2)	3.09	(0.40, 62.4)	0.33
Cord blood	26/294 (8.8)	23/299 (7.7)	1.15	(0.66, 2.03)	0.63

RR – risk ratio. MD – mean difference. IV – intravenous. Hypophosphataemia refers to phosphate levels < 0.8075 mmol/l in maternal blood for the first 4 time points and phosphate level <1.4mmol/l for cord blood.

Figure SM1

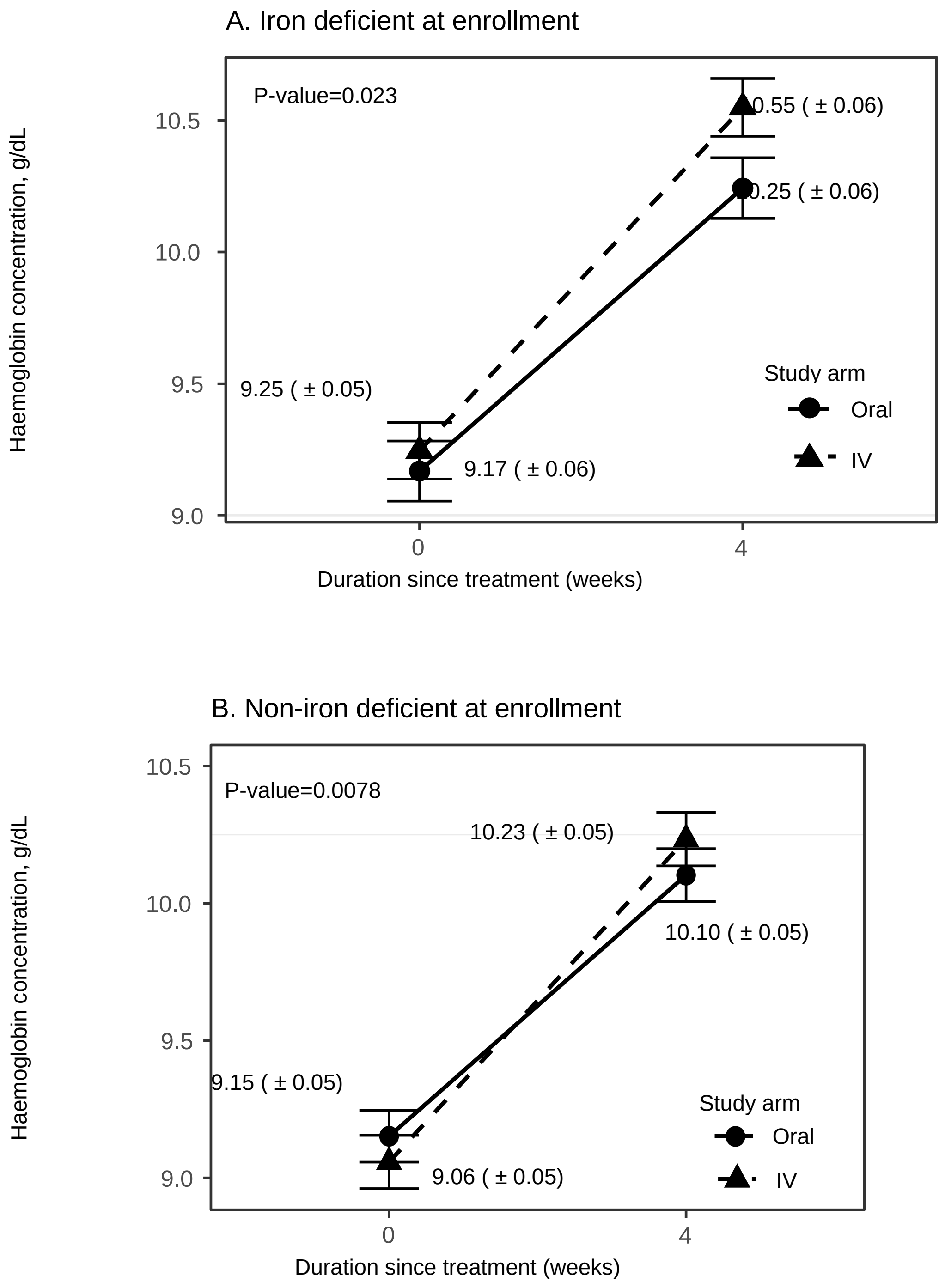

Figure SM1. Treatment effect on the increase in maternal haemoglobin (n = 1,056).

Intravenous versus oral iron for iron deficiency anemia in pregnant Nigerian women (IVON): an open label, randomized controlled trial: Statistical Analysis Plan

SAP Signatures

I give my approval for the attached SAP entitled "Intravenous versus oral iron for iron deficiency anemia in pregnant Nigerian women (IVON): an open-label, randomized controlled trial" dated 21st July 2023.

Statistician (Author)
Name: Dr. Ibraheem Abioye

Signature: Ibraheem Abioye

Date: 21 July 2023

Principal Investigator (optional)
Name: Prof. Bosede B. Afolabi

Signature: Afolabi

Date: 21 July 2023

Table of Contents

SAP Signatures 7

Table of Contents 8

Abbreviations and Definitions 10

1 Introduction 11

1.1 Preface 11

1.2 Scope of the analyses 11

2 Study Objectives and Endpoints 11

2.1 Study Objectives 11

2.2 Endpoints 12

3 Study Methods 13

3.1 General Study Design and Plan 13

3.2 Inclusion-Exclusion Criteria and General Study Population 13

3.3 Randomization and Blinding 14

3.4 Study Assessments 14

Analysis Time Windows 15

Description of variables 15

4 Sample Size 16

5 General Analysis Considerations 17

5.1 Timing of Analyses 17

5.2 Analysis Populations 17

5.2.1 Intention to Treat (ITT) population 17

5.2.2 Modified Intention to Treat (mITT) population 17

5.2.3 Per protocol population 17

5.3 Covariates and Subgroups 17

5.3.1 Analysis by region 18

5.4 Missing Data 18

5.5 Multiple Testing 18

6 Summary of Study Data 18

6.1 Subject Disposition 19

Follow-Up 19

Analysis 19

Enrollment 19

Allocation 19

6.2 Derived variables 20
6.3 Protocol Deviations 21
6.4 Concurrent Illnesses and Medical Conditions 21
6.5 Treatment Compliance 21
7 Efficacy Analyses 21
7.1 Co-primary Efficacy Analysis – anaemia at 36 weeks' gestation 21
7.2 Co-primary Efficacy Analysis – preterm birth 22
7.3 Secondary Efficacy Analyses 22
7.3.1 Secondary Analyses of Primary Endpoints 22
7.3.2 Analyses of Important Secondary Endpoint – maternal depression 22
7.3.3 Analyses of Secondary Endpoints 23
7.4 Subgroup analyses 23
8 Safety Analyses 23
8.1 Extent of Exposure 23
8.2 Serious Adverse Events (SAE) and other Significant Adverse Events 24
9 Reporting Conventions 24
10 Quality Assurance of Statistical Programming 24
11 Summary of Changes to the Protocol and/or SAP 24
Rationale for Adjustments of Statistical Analysis Plan from Protocol 25
12 References 25
13 Listing of Tables, Listings and Figures 26

Abbreviations and Definitions

AE	Adverse Event
AE(s)	Adverse Event(s)
CRF	Case Report Form
IDA	Iron deficiency anemia
SAP	Statistical Analysis Plan

1 Introduction

1.1 Preface

Anaemia in pregnancy (AIP) is common in many low- and middle- income countries (LMICs) including Nigeria. It leads to substantial or life-threatening maternal and infant complications which can potentially be prevented if anaemia is promptly and adequately treated. Iron deficiency is the commonest cause of AIP; in LMICs, its treatment is typically by oral iron, which is often-poorly tolerated and not fully complied with.

Intravenous iron requires minimal patient-facility contact and corrects anaemia much faster than oral preparations. Recent intravenous iron preparations have been found to be well tolerated and with fewer adverse effects than the previously available high molecular weight iron dextrans.

In the Nigerian setting, pregnant women seek antenatal care late, and have poor ANC clinic attendance. Thus, the use of a minimally dosed iron formulation that is safe, rapidly effective, and cost-effective can improve the likelihood of prompt and appropriate IDA treatment and potentially reduce the risk of complications.

Prior evidence has not shown oral iron to be impactful for important clinical outcomes such as low birthweight, preterm delivery; thus, intravenous iron may be more effective in this regard. Findings from this study could potentially protect significant proportions of pregnant women and neonates in LMICs from severe morbidity and mortality.

1.2 Scope of the analyses

These analyses will assess the effectiveness and safety of intravenous ferric carboxymaltose in comparison to oral ferrous sulphate (control) to treat iron deficiency anaemia (IDA) and will be included in the clinical study report.

2 Study Objectives and Endpoints

2.1 Study Objectives

To determine the comparative effectiveness of intravenous ferric carboxymaltose (intervention) versus oral ferrous sulphate (control) for treating iron deficiency anaemia in pregnancy and to compare the tolerability, safety, and the cost-effectiveness of intravenous versus oral iron among pregnant Nigerian women with moderate and severe IDA at 20-32 weeks' gestation.

Specific objectives include:
1. To determine the effect of intravenous ferric carboxymaltose on the prevalence of maternal anaemia at 36 weeks' gestation and on the increase in haemoglobin concentration 4 weeks after administration compared with oral ferrous sulphate in pregnant women with iron deficiency anemia.
2. To determine the effect of intravenous ferric carboxymaltose on the incidence of postpartum haemorrhage, sepsis, shock, the need for blood transfusion, the prevalence of depression and other maternal clinical outcomes, compared with oral ferrous sulphate in pregnant women with iron deficiency anaemia.
3. To determine the effect of intravenous ferric carboxymaltose on the incidence of low infant birthweight, prematurity, stillbirth, and neonatal mortality, and on breastfeeding and immunization, compared with the use of oral ferrous sulphate in pregnant women with iron deficiency anaemia.
4. To measure implementation outcomes of intravenous ferric carboxymaltose including its acceptability, feasibility, and fidelity in the context in which the trial is being carried out.
5. To determine the cost-effectiveness of intravenous ferric carboxymaltose compared with oral ferrous sulphate in the treatment of iron deficiency anaemia in pregnancy.

2.2 Endpoints

Primary

1. Prevalence of maternal anaemia at 36 weeks
   The prevalence of maternal anaemia is defined as haemoglobin <10g/dL at 36 weeks. Haemoglobin measurement nearest to 36 + 0 weeks of gestation will be used, of all the tests between 30 weeks and delivery. Analysis will be by intention to treat, using log-binomial models that allow the estimation of risk ratios, 95% confidence intervals and two-tailed p-values. No covariate adjustment will be considered. Missing values will be ignored.
2. Incidence of preterm birth
   Preterm births will be defined as births before 37 + 0 weeks gestation using the agreed gestational age at trial entry. Analysis will be by intention to treat, using log-binomial models that allow the estimation of risk ratios, 95% confidence intervals and two-tailed p-values. No covariate adjustment will be considered. Missing values will be ignored.

Hypothesis:
1. We expect a 14% lower prevalence of anaemia at 36 weeks' gestation in the ferric carboxymaltose (intervention) group compared to the ferrous sulphate (control) group.
2. There will be a lower incidence of preterm birth among the intervention group, compared with the control group.

Secondary

1. Increase in maternal haemoglobin levels at 4 weeks post-initiation of treatment. *In some cases, samples included in the 36-week primary endpoint analysis will also be included here if they meet both criteria.*

2. The safety and tolerability of intravenous ferric carboxymaltose versus oral ferrous sulphate, including the incidence of hypophosphatemia and severity of maternal adverse effects.

3. Severe maternal events, specifically, postpartum haemorrhage, sepsis, shock, and the need for blood transfusion.

4. The incidence of

a. low infant birthweight (<2.5 kg),

b. prematurity (<37 weeks' gestation as dated from the last menstrual period or early ultrasound scan done not later than 22 weeks gestational age if unsure of LMP) (25)

c. stillbirth and,

d. neonatal mortality (birth till 28 days of life),

5. Proportion of infants

    a. being breastfed at 2 and 6 weeks of life, and

    b. having received vaccines up-to-date (BCG, oral polio and hepatitis vaccination) in same time period.

6. The incidence of small for gestational age (birthweight less than the 10th percentile for gestational age).

7. Incidence of depression linked to emotional well-being of mothers using the validated Edinburgh Postnatal Depression Scale (EPDS).

    Depression will be defined as EPDS score >10 any time after birth. Given the EPDS is assessed multiple times, the highest score will be used. Any woman who commits or attempts suicide will be regarded as depressed, regardless of her score. Any woman who self-reports depressive illness or whose family member or physician reports a depressive illness will also be regarded as depressed.

    Analysis will be by intention to treat, using log-binomial models that allow the estimation of risk ratios, 95% confidence intervals and two-tailed p-values. No covariate adjustment will be considered. Missing values will be ignored.

3 Study Methods

3.1 General Study Design and Plan

Multicenter, parallel, open label individually randomized controlled trial, with 1,056 women allocated in a 1:1 ratio in conjunction with a cost-effectiveness analysis.

Single dose of 20mg/kg IV ferric carboxymaltose (not exceeding 1000mg). Intravenous route

Daily administration of 200mg (65mg elemental iron) 3 times daily oral ferrous sulphate. Oral route.

Participants will be seen in clinic every 4 weeks till 28 weeks' gestation and every 2 weeks until 36 weeks, then weekly until delivery.

3.2 Inclusion-Exclusion Criteria and General Study Population

Inclusion criteria:

- Pregnant women aged 15 to 49 years old between 20*- and 32**-weeks' gestational age
  - 20 weeks was chosen as lower limit because Nigerian women register for ANC care in the second trimester, typically at 20 weeks or later.
  - 32 weeks as the upper limit to enable assessment of impact of both intervention and standard of care on perinatal events by evaluating their haemoglobin concentration by 36 weeks.
- Baseline (enrollment) laboratory-confirmed moderate or severe anaemia (Hb < 10g/dl).

Exclusion criteria:
- Medically confirmed significant bleeding, major surgery or received blood transfusion within the last 3 months.
- Severe symptomatic anaemia needing urgent correction with blood transfusion.
- Anaemia of other known causes besides IDA e.g., sickle cell anaemia, thalassemia, autoimmune diseases, chronic kidney disease, cancer, human immunodeficiency virus infection (HIV).
- Clinically confirmed malabsorption syndrome
- Hypersensitivity to any form of iron treatment.
- History of any immune related illness e.g., SLE, Rheumatoid arthritis
- Preexisting maternal depression or other psychiatric illness
- Severe allergic reactions such as severe asthma
- History of known drug allergy

3.3 Randomization and Blinding

At the enrolment visit, a pregnant woman who is found to have AIP through haemoglobin testing, using the Hemocue® haemoglobinometer, with a haemoglobin concentration of 9.9g/dL or lower, who meets the eligibility criteria and gives informed consent will be enrolled. Eligible participants will be consecutively enrolled. They will be randomized to one of the two treatments groups. Individual randomization and allocation concealment will be done with the use of a web-based randomization software known as 'Sealed envelope' in a 1:1 ratio in blocks stratified according to center

3.4 Study Assessments

Table 1. Schedule of study assessments

Visit	Treatment (Baseline)	4 weeks' post-enrollment	36 weeks' EGA	Delivery	2 wks pp	6 wks pp
**Socio-demographics**	X					
**Physical exam**	X	X	X	X	X	X
**Haemoglobin**	X	X	X	X	X	X
**Malaria**	X					
**FBC**	X + 4 weeks after	X	X	X		X
**Iron panel**	X + 4 weeks after	X	X	X		X
**Maternal serum PO_4**	X + 4 weeks after	X	X	X		X
**Cord blood PO_4**				X		

**EPDS**	X	X	X		X	
**Adverse events**	X	X	X	X	X	X
**Child immunization status**						X

Analysis Time Windows

We will allow the inclusion of variables collected around the following time windows.

Table 2. Analysis Time Windows

Visit (target day)	Lower bound (days)	Upper bound (days)
**Baseline (0)**	N/A	N/A
**36 weeks EGA**	-6	Any time before delivery
**Delivery**	0	+2
**2 wks pp**	10	18
**4 wks pp**	19	34
**6 wks pp**	35	49

Description of variables

The key variables used for analysis are described below;

Table 3. Description of variables

Variable	Description
**Haemoglobin**	Continuous variable, measured in g/dL. Usually, the lower limit of the measured range is 3 g/dL and the upper limit is 20 g/dL.
**Anaemia**	Calculated from haemoglobin variable, <10g/dL<br>▪ The first primary endpoint is anaemia at 36 weeks' gestation
**Gestational age at birth**	Continuous variable, measured in weeks. The lower limit of acceptable range is 20 weeks. The usual upper limit is 44 weeks, beyond which baby is unlikely to have survived.
**Preterm birth**	Measured from gestational age at birth<br>▪ The first second primary endpoint is preterm birth
**Serum phosphate**	Continuous variable, measured in mmol/L.
**Hypophosphatemia**	Measured from serum phosphate <0.8075 mmol/L (equivalent to 2.5 mg/dL) (1)<br>▪ A secondary endpoint
**Haemorrhage**	Binary variable (0, 1). Bleeding during pregnancy or postpartum
**Sepsis**	Binary variable (0, 1). As defined by clinician
**Shock**	Binary variable (0, 1). As defined by clinician
**Need for blood transfusion**	Binary variable (0, 1). As defined by clinician
**Incidence of severe maternal events**	Determined based on the incidence of any of haemorrhage, sepsis, shock and need for blood transfusion<br>▪ A secondary endpoint

Variable	Description
**Birthweight**	Continuous variable, measured in grams, rounded to every 10g
**Low birthweight**	Measured from birthweight<br>▪ A secondary endpoint
**Stillbirth**	Binary variable (0, 1). Gestational age must be ≥28 weeks, the age of viability.<br>▪ A secondary endpoint
**Neonatal mortality**	Binary variable (0, 1). Defined as infant age at death <42 days<br>▪ A secondary endpoint
**Breastfed infants at 1, 2 and 4 weeks**	Binary variable (0,1).<br>▪ A secondary endpoint
**BCG vaccination**	Binary (0,1) at 1, 2 and 4 weeks
**Oral polio vaccination**	Binary (0,1) at 1, 2 and 4 weeks
**Hepatitis vaccination**	Binary (0,1) at 1, 2 and 4 weeks
**Vaccination up-to-date**	Binary (0,1) at 1, 2 and 4 weeks. Calculated from BCG, oral polio and hepatitis vaccination.<br>▪ A secondary endpoint
**Small for gestational age (SGA)**	Binary variable (0,1). Calculated from the birthweight and gestational age based on the Oken thresholds(1).
**EPDS score**	Continuous variable.
**Depression**	Binary variable (0,1). Calculated from the EPDS score. Depression will be defined as EPDS score >10 any time after birth. Given the EPDS is assessed multiple times, the highest score will be used. Any woman who commits or attempts suicide will be regarded as depressed, regardless of her score. Any woman who self-reports depressive illness or whose family member or physician reports a depressive illness will also be regarded as depressed.

4 Sample Size

At the 5% significance and precision level, **1,056 pregnant women** (528 in each study arm) are required to detect a difference in improvement in the prevalence of AIP at term by 14%, between the control group (70% corrected) and the intervention group (84% corrected), as seen in a multi-country international study in Europe, Asia and Australia(2) at 90% power, adjusting for 15% attrition and protocol violations(3).

To assess the outcome of increase in haemoglobin concentration: At the 5% significance level, **990 pregnant women** (495 in each study arm) are required to detect a difference in improvement in the Hb level after 4 weeks among anaemic pregnant women at term by 1g/l, between the control group and the intervention group, at 90% power, adjusting for a 15% attrition and protocol violations, giving a superiority and two-tailed tests of hypotheses(3). A systematic review reported a pooled confidence interval of mean difference of haemoglobin between treatment and control arm as 3.9 to +10.9 g/L(4) while Kochhar et al. in India(5) reported a difference in mean haemoglobin of 2g/dl. We therefore assumed a conservative clinically relevant effect size of 1g/dl to achieve the current sample size.

There was no previous study in our environment describing the efficacy of intravenous iron administration on the outcome of preterm deliveries among pregnant women with anaemia. Prevalence of preterm birth in Nigeria is between 16.8% and 32.9%(6-8). According to a systematic review, there was about 1.6-fold risk of preterm delivery among anaemic mothers. (RR: 1.56, 95%CI: 1.25 – 1.95)(9). Thus, the prevalence of preterm delivery among anaemic mothers is assumed to be between 28.9% and 52.6%.

Hence, we utilized the power calculator in Stata version 17 statistical software (StataCorp. 2021. *Stata Statistical Software: Release 17*. College Station, TX: StataCorp LLC) (40) to calculate the minimum sample size to be 892 women (446 per arm) based on the assumption that prevalence of preterm deliveries among anaemic pregnant women was 28.9%, given 90% power, a protective relative risk of intravenous iron against preterm delivery of 0.65 and a 20% loss to follow -up.

To assess the secondary outcome of prevalence of low birth weight: At the 5% significance level, **892 pregnant women** (446 in each study arm) are required to detect a 20% decrease (as shown from prenatal iron use)(10) in the prevalence of low birth weight from 15% (average in Nigeria) to 12%, at the 5% significance level with 90% power, adjusting for a 15% attrition and protocol violations.

For depression, using a 13% incidence of post-partum depression (PPD) among non-anemic patients, a sample size of **294 pregnant women** (147 women per arm) would detect a 2.8-fold increase in PPD (37%) or higher in the defined anemic group (hemoglobin < 110)(11), with a statistical power of 90% and a 5% significance level, while adjusting for a 15% attrition rate.

A total of 1056 pregnant women with GA between 20 and 32 weeks will be enrolled into the IVON study.

5 General Analysis Considerations

5.1 Timing of Analyses

The final analysis will be performed on the final unblinded dataset, after data cleaning is completed and database is locked.

5.2 Analysis Populations

5.2.1 Intention to Treat (ITT) population

- The intention to treat population refers to all subjects who were randomized. Following the intention-to-treat principle, patients will be analyzed according to the treatment they were assigned to at randomization.

5.2.2 Modified Intention to Treat (mITT) population

- All ITT patients that completed 36 weeks or delivery will be eligible for assessment of the primary endpoint. This population will be considered in exploratory analysis.

5.2.3 Per protocol population

- A per protocol population will not be considered in the analysis

5.3 Covariates and Subgroups

The following table is a list of covariates to be presented in Table 1.

Table

Variable	Description
Age	Continuous variable, measured in years
Age categories	Categorical variable
Haemoglobin	Continuous variables, g/dL
Anaemia categories	Moderate, severe
Marital status	Married, not married
Place of residence	Rural, urban
Ethnicity	Hausa, Igbo, Yoruba, Others
Socioeconomic status	Upper, Middle, Lower
Infant sex	Dichotomous variable (male, female)
Center	Categorical variable
Region	Categorical variable
Haemoglobin at baseline	Continuous variable
Anemia categories at baseline	Categorical variable, based on WHO classification

Additional baseline variables to be considered. Socioeconomic status is to be defined based on the educational status and occupation following the Ibadin classification(12).

5.3.1 Analysis by region

The frequency of key covariates and endpoints will be evaluated across regions. In the main analysis, the analysis will be conducted without evaluating effects by the region. In exploratory analysis, subgroup analysis will be done by region and will be presented if significant differences exist.

5.4 Missing Data

No imputation of endpoints will be done in the main analysis.

Missingness of covariates will only be considered in the sensitivity analysis. The frequency of missingness in the endpoints and key covariates will be assessed and presented using bar graphs. If ≤2 key covariates are missing >5% of observations, inverse probability weighting will be used to address missingness during sensitivity analysis(13). If >2 covariates are missing >5% of covariates, multiple imputation will be used to address missingness in analysis(14).

5.5 Multiple Testing

All p-values will be presented to the third decimal place. There are two co-primary endpoints in our analysis. The Bonferroni method will be used to adjust the nominal significance level(15). Thus, the alpha level for statistical inference in our analysis will be 0.025.

6 Summary of Study Data

For the ITT populations, baseline covariates will be summarized to describe the population.

Continuous variables will be summarized using the following descriptive statistics, n (non-missing sample size), mean, standard deviation (SD), medians, minimum and maximum. The frequency and percentages (based on the non-missing sample size) of observed levels will be reported for all categorical measures. In general, all summary tables will be structured with a column for the overall population and sorted by region. Summary tables will also be presented for each treatment, and will be annotated with the total population size relevant to that table/treatment. The number of missing observations will be presented in the footnote of each table.

6.1 Subject Disposition

The following CRFs will be used to determine which participants reached the following stages.

Visit (target day)	CRF
**Baseline (0)**	Enrolment form
**Delivery**	Delivery form
**2 wks pp**	2-weeks post-partum form
**6 wks pp**	6-weeks post- [illegible]

The time-dependent rates of recruitment will be provided in graphical format.

A flow diagram of participant selection will be provided as below to provide an explicit statement of the key statistics of the study.

Figure. Flow diagram

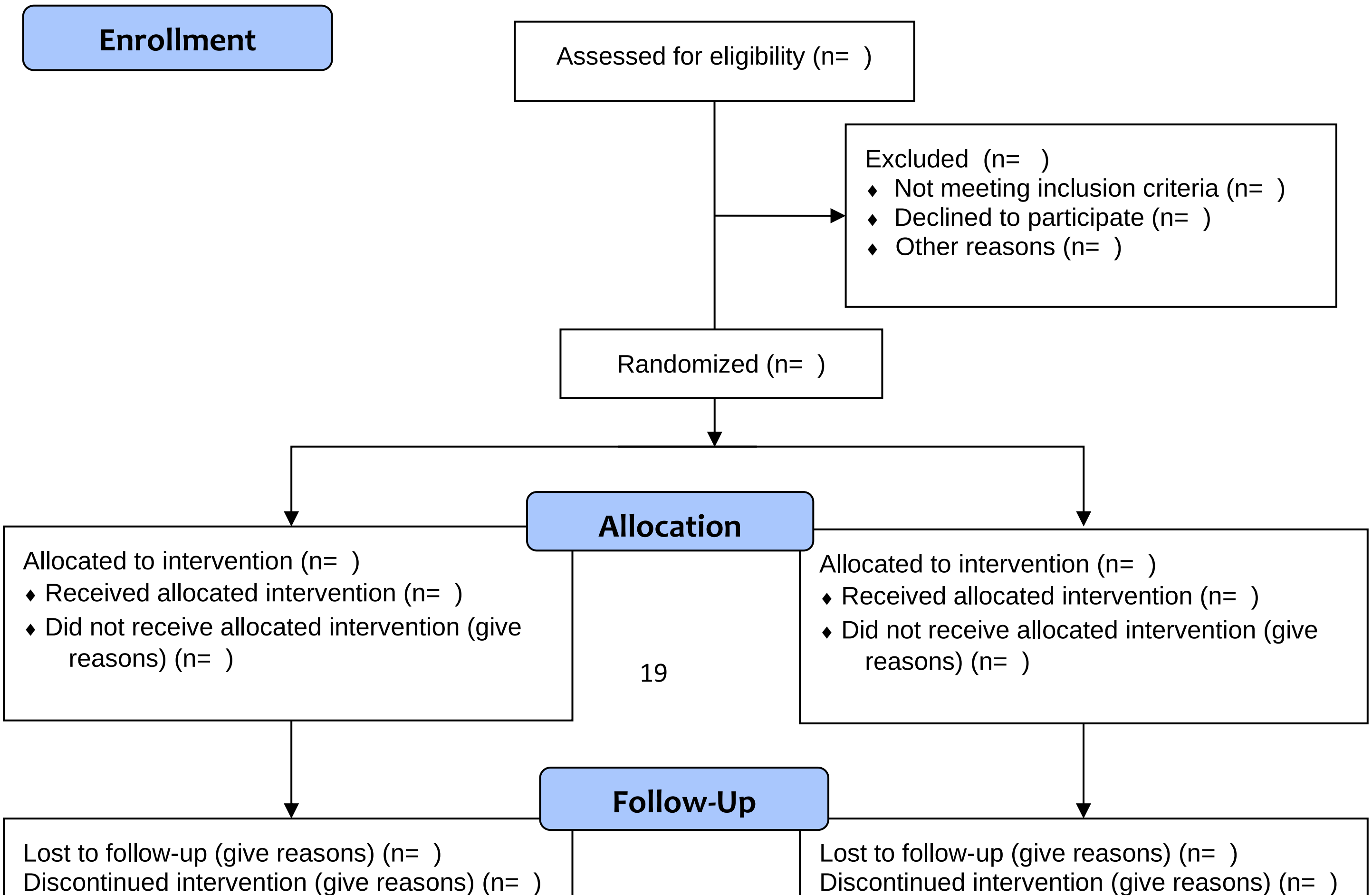

6.2 Derived variables

Anemia and preterm birth are primary endpoints that are derived variables. A number of secondary endpoints are also derived variables. Their definitions are provided in the table below.

Variable	Description
**Anaemia**	Calculated from haemoglobin variable, <10g/dL<br>▪ The first primary endpoint is anaemia at 36 weeks' gestation
**Iron deficiency anaemia (IDA)**	Calculated from haemoglobin <10g/dL and ferritin <30 µg/L<br>▪ IDA at baseline will be the basis for subgroup analyses
**Preterm birth**	Measured from gestational age at birth<br>▪ The first second primary endpoint is preterm birth
**Hypophosphatemia**	Measured from serum phosphate <0.8075 mmol/L (equivalent to 2.5 mg/dL) (1)<br>▪ A secondary endpoint
**Low birthweight**	Measured from birthweight<br>▪ A secondary endpoint
**Stillbirth**	Binary variable (0, 1). Gestational age must be ≥28 weeks, the age of viability.<br>▪ A secondary endpoint
**Postpartum haemorrhage**	Binary variable (0, 1). Blood loss postpartum > 1,000 ml based on visual or weight method, whichever is greater(16).
**Neonatal mortality**	Binary variable (0, 1). Defined as infant age at death <42 days<br>▪ A secondary endpoint
**Vaccination up-to-date**	Binary (0,1) at 1, 2 and 4 weeks. Calculated from BCG, oral polio and hepatitis vaccination.<br>▪ A secondary endpoint
**Small for**	Binary variable (0,1). Calculated from the birthweight and gestational age.

Variable	Description
gestational age (SGA)	
EPDS score	Continuous variable. Individuals identified as depressed despite EPDS being <10 will have their EPDS corrected to 10/median EPDS for the depressed subgroup
Depression	Binary variable (0,1). Calculated from the EPDS score. Depression will be defined as EPDS score >10 any time after birth. Given the EPDS is assessed multiple times, the highest score will be used. Any woman who commits or attempts suicide will be regarded as depressed, regardless of her score. Any woman who self-reports depressive illness or whose family member or physician reports a depressive illness will also be regarded as depressed.

6.3 Protocol Deviations

Given that analysis will be by ITT or mITT, no specific protocol deviations will impact the approach to analysis. The summary statistics will be produced in accordance with section 5 (General Analysis Considerations).

6.4 Concurrent Illnesses and Medical Conditions

The summary statistics of any concurrent illnesses and medical conditions will be produced in accordance with section 5 (General Analysis Considerations).

6.5 Treatment Compliance

Treatment compliance was assessed using the remaining pill count and diary records. Each participant's average compliance rate will be estimated thus:

$$\frac{Number\ of\ pills\ absent\ from\ returned\ regimen\ bottles}{Number\ of\ days\ participant\ had\ the\ bottle}\%$$

The summary statistics will be produced in accordance with section 9.

7 Efficacy Analyses

7.1 Co-primary Efficacy Analysis – anaemia at 36 weeks' gestation

The main analysis will be conducted in the ITT population. We expect a 14% lower prevalence of anaemia at 36 weeks' gestation in the ferric carboxymaltose (intervention) group compared to the ferrous sulphate (control) group. The null hypothesis is that there is no difference in the prevalence of anaemia at 36 weeks' gestation between the intervention and control groups.

The frequency of occurrence of the two categorical primary endpoints will be presented as N and percent of the total study population, by region, and by treatment group (IV iron vs. oral iron). Log-binomial regression models will be used and risk ratios and confidence intervals presented.

To obtain the relative risk of anemia accounting for region and facility type, logistic generalized

linear mixed regression models will be used, and the beta coefficients exponentiated(17). Relevant measures of uncertainty (confidence intervals and p-values) will be reported. In addition, log-binomial regression models with and without statistical control for region and facility type will be estimated and compared with the log-binomial GLMM model. The final model will be selected using the Akaike Information Criterion (AIC). In some cases, the log-binomial models may not converge and log-Poisson models, which provide consistent but not fully efficient estimates of the relative risk, and its confidence intervals will be used(18). Results will be presented in figures.

7.2 Co-primary Efficacy Analysis – preterm birth

The main analysis will be conducted in the ITT population.

The incidence of preterm births will be presented as N and percent of the total study population, by region, and by treatment group (IV iron vs. oral iron).

Hypothesis:

1. There will be a lower incidence of preterm birth among the intervention group, compared with the control group.

Log-binomial regression models will be used, and risk ratios and confidence intervals presented.

To obtain the relative risk of preterm birth accounting for region and facility type, logistic generalized linear mixed regression models will be used, and the beta coefficients exponentiated(17). Relevant measures of uncertainty (confidence intervals and p-values) will be reported. In addition, log-binomial regression models with and without statistical control for region and facility type will be estimated and compared with the log-binomial GLMM model. The final model will be selected using the Akaike Information Criterion (AIC). In some cases, the log-binomial models may not converge and log-Poisson models, which provide consistent but not fully efficient estimates of the relative risk, and its confidence intervals will be used(18). Results will be presented in figures.

7.3 Secondary Efficacy Analyses

7.3.1 Secondary Analyses of Primary Endpoints

The mITT population will be used for analysis. The maternal anaemia and preterm birth analyses will be repeated and findings compared to the primary analyses.

7.3.2 Analyses of Important Secondary Endpoint – maternal depression

This analysis will be conducted in the ITT populations.

The EPDS score will summarized as a continuous outcome using N, mean, standard deviation (SD), median, minimum and maximum, overall and by treatment group, at baseline and 36 weeks. The proportion of participants that attain the minimally important change of four points will be estimated and compared by treatment group(19).

The frequency of occurrence of maternal depression will be presented as N and percent of the total study population, by region, and by treatment group (IV iron vs. oral iron).

To obtain the relative risk and 95% CI of maternal depression, as well as the relative risk of achieving the minimally important change, log-binomial regression models will be used. In some cases, the log-binomial models may not converge and log-Poisson models, which provide consistent but not fully efficient estimates of the relative risk, and its confidence intervals will be used(18).

7.3.3 Analyses of Secondary Endpoints

This analysis will be conducted in both the ITT and mITT populations.

The frequency of occurrence of the categorical endpoints will be presented as N and percent of the total study population, by region, and by treatment group (IV iron vs. oral iron). N, mean, standard deviation (SD), median, minimum and maximum will summarize continuous variables.

To obtain the relative risk and 95% CI of the occurrence of the primary and secondary endpoints, log-binomial regression models will be used. In some cases, the log-binomial models may not converge and log-Poisson models, which provide consistent but not fully efficient estimates of the relative risk, and its confidence intervals will be used(18). Results will be presented in tables and figures.

7.4 Subgroup analyses

The ITT population will be used for analysis. The outcomes of interest will be the primary and secondary endpoints. The analyses will be conducted among those with iron deficiency anaemia at enrolment compared to those without.

Continuous endpoints will summarized using N, mean, and standard deviation (SD), in the overall IDA (vs. non-IDA population) and by treatment group, at 36 weeks. Linear regression models will be used to obtain mean difference and 95% CI in each subgroup.

The frequency of occurrence of the dichotomous endpoints will be presented as N and percent of the IDA (vs. non-IDA) population, and by treatment group (IV iron vs. oral iron).

To obtain the relative risk and 95% CI of each dichotomous endpoint, log-binomial regression models will be used. In some cases, the log-binomial models may not converge and log-Poisson models, which provide consistent but not fully efficient estimates of the relative risk, and its confidence intervals will be used(18). Likelihood ratio tests will be used to compare models with an interaction term for IDA status and treatment to those without.

8 Safety Analyses

8.1 Extent of Exposure

We will examine the time to occurrence of any serious adverse events that occur in >5% of individuals who receive either intervention. This will be presented using median time to event as well as graphically with Kaplan-Meier curves.

8.2 Serious Adverse Events (SAE) and other Significant Adverse Events

The number and proportion of participants who experience serious adverse events that are known to be related to the treatment effect will be analysed and presented. The appropriate grading of severity of the SAEs will also be presented in counts and proportion.

The following SAEs will be considered at the minimum, though additional

9 Reporting Conventions

P-values ≥0.001 will be reported to 3 decimal places; p-values less than 0.001 will be reported as "<0.001". The mean, standard deviation, and any other statistics other than quantiles, will be reported to one decimal place greater than the original data. Quantiles, such as median, or minimum and maximum will use the same number of decimal places as the original data. Estimated parameters, not on the same scale as raw observations (e.g. regression coefficients) will be reported to 3 significant figures.

10 Quality Assurance of Statistical Programming

A second review statistician will independently reproduce the primary analyses, and summary statistics table X, Y, Z. The reviewing statistician will have an overview of the entire analyses and will explicitly check the code producing tables (selected at random) as well as any other pieces of code as desired.

To provide high quality code that is understandable, and allows reproduction of the analysis the following points will be followed.

The population to be used in a table or figure will be explicitly set at the start of a block of code that computes the output, ideally by looking up the population from the table of tables.

Any outputs will have the

- date and time included
- the name of the code file that produced the analysis
- the author

At the start of any code file there will be a set of comments that give

- the author
- the date and time of writing
- references to inputs and outputs
- reference to any parent code file that runs the child code file

11 Summary of Changes to the Protocol and/or SAP

Rationale for Adjustments of Statistical Analysis Plan from Protocol

Any changes from the protocol-specified definitions of aims, outcomes and statistical analytic approaches will be outlined below. These represent changes made prior to the database lock and unblinding of the study.

a) Subgroup analysis based on iron deficiency anaemia was included.
   - Earlier versions of the protocol had prespecified this analysis, which was omitted in error from the final version.
b) Iron deficiency analysis at 36 weeks was added as a secondary endpoint
   - This endpoint was included to improve the evaluation of hematologic outcomes in the study
c) Instead of evaluating the prevalence of vaccination with each of BCG, OPV and HBV, the secondary endpoint was changed to whether vaccination was up-to-date for baby's age.
d) Breastfeeding timepoints for assessment were reduced to 2 and 6 weeks to align with study visits.
e) The threshold for defining anaemia was changed from 11 g/dL to 10 g/dL which is in the published protocol.

12 References

1. Sharma S, Hashmi MF and D. C. Hypophosphatemia. *StatPearls*. Treasure Island (FL), StatPearls Publishing, 2022.

2. Breymann C, Milman N, Mezzacasa A, Bernard R, Dudenhausen J and investigators F-A. Ferric carboxymaltose vs. oral iron in the treatment of pregnant women with iron deficiency anemia: an international, open-label, randomized controlled trial (FER-ASAP). Journal of perinatal medicine. 2017; 45:443-53.

3. Bolarinwa OA. Sample size estimation for health and social science researchers: the principles and considerations for different study designs. Nigerian Postgraduate Medical Journal. 2020; 27:67.

4. Qassim A, Grivell RM, Henry A, Kidson-Gerber G, Shand A and Grzeskowiak LE. Intravenous or oral iron for treating iron deficiency anaemia during pregnancy: systematic review and meta-analysis. Medical Journal of Australia. 2019; 211:367-73.

5. Kochhar PK, Kaundal A and Ghosh P. Intravenous iron sucrose versus oral iron in treatment of iron deficiency anemia in pregnancy: a randomized clinical trial. Journal of Obstetrics and Gynaecology Research. 2013; 39:504-10.

6. Bello M, Pius S and Ibrahim BA. Characteristics and predictors of outcome of care of preterm newborns in resource constraints setting, Maiduguri, Northeastern Nigeria. Journal of Clinical Neonatology. 2019; 8:39.

7. Azeez B, Chinyere E, Osayame E, et al. Characteristics and risk factors of preterm births in a tertiary center in Lagos, Nigeria. The Pan African Medical Journal. 2016; 24.

8. Olusanya BO and Ofovwe GE. Predictors of preterm births and low birthweight in an inner-city hospital in sub-Saharan Africa. Maternal and child health journal. 2010; 14:978-86.

9. Rahmati S, Azami M, Badfar G, Parizad N and Sayehmiri K. The relationship between maternal anemia during pregnancy with preterm birth: a systematic review and meta-analysis. The Journal of Maternal-Fetal & Neonatal Medicine. 2020; 33:2679-89.

10. Haider BA, Olofin I, Wang M, Spiegelman D, Ezzati M and Fawzi WW. Anaemia, prenatal iron use, and risk of adverse pregnancy outcomes: systematic review and meta-analysis. Bmj. 2013; 346.

11. Chandrasekaran N, De Souza LR, Urquia ML, et al. Is anemia an independent risk factor for postpartum depression in women who have a cesarean section?-A prospective observational study. BMC Pregnancy and Childbirth. 2018; 18:1-7.

12. Ibadin MO and Akpede GO. A revised scoring scheme for the classification of socio-economic status in Nigeria. Nigerian Journal of Paediatrics. 2021; 48:26-33.
13. Seaman SR and White IR. Review of inverse probability weighting for dealing with missing data. Statistical methods in medical research. 2013; 22:278-95.
14. van Buuren S, Groothuis-Oudshoorn K, Robitzsch A, Vink G, Doove L and Jolani S. Package 'mice'. Computer software. 2015.
15. Li G, Taljaard M, Van den Heuvel ER, et al. An introduction to multiplicity issues in clinical trials: the what, why, when and how. International journal of epidemiology. 2017.
16. Wormer KC, Jamil RT and SB. B. Acute Postpartum Hemorrhage. *StatPearls*. Treasure Island (FL), StatPearls Publishing, 2022.
17. Pedroza C and Truong VTT. Estimating relative risks in multicenter studies with a small number of centers—which methods to use? A simulation study. Trials. 2017; 18:1-10.
18. Zou G. A modified poisson regression approach to prospective studies with binary data. American Journal of Epidemiology. 2004; 159:702-6.
19. Mao F, Sun Y, Wang J, Huang Y, Lu Y and Cao F. Sensitivity to change and minimal clinically important difference of Edinburgh postnatal depression scale. Asian Journal of Psychiatry. 2021; 66:102873.

13 Listing of Tables, Listings and Figures

TBC
