# Supplementary material for: Intravenous versus oral iron for anaemia among pregnant women in Nigeria (IVON): an open-label, randomised controlled trial
Source: Lancet Glob Health. 2024 Sep 18;12(10):e1649–59. doi: 10.1016/S2214-109X(24)00239-0 (PMC11420468; doi:10.1016/S2214-109X(24)00239-0)
Supplement: Equitable Partnership Declaration [file mmc2.pdf]

THE LANCET
Global Health

Supplementary appendix 2

This Equitable Partnership Declaration (EPD) was submitted by the authors, and we reproduce it as supplied. It has not been peer reviewed. *The Lancet*'s editorial processes have not been applied to the EPD.

Supplement to: Afolabi BB, Babah OA, Adeyemo TA, et al. Intravenous versus oral iron for anaemia among pregnant women in Nigeria (IVON): an open-label, randomised controlled trial. *Lancet Glob Health* 2024; **12:** e1649–59.

Equitable Partnership Declaration questions

Researcher considerations

1. Please detail the involvement that researchers who are based in the region(s) of study had during a) study design; b) clinical study processes, such as processing blood samples, prescribing medication, or patient recruitment; c) data interpretation; and d) manuscript preparation, commenting on all aspects. If they were not involved in any of these aspects, please explain why.

   This question is intended for international partnerships; if all your authors are based in the area of study, this question is not applicable.

   This should include a thorough description of their leadership role(s) in the study. Are local researchers named in the author list or the acknowledgements, or are they not mentioned at all (and, if not, why)? Please also describe the involvement of early career researchers based in the location of the study. Some of this information might be repeated from the Contributors section in the manuscript. Note: we adhere to ICMJE authorship criteria when deciding who should be named on a paper.

**a) Study design:**<br>The research idea was conceptualized by BBA, JT, OAB, TAA, MB, HSG, NASA, and GO. The design of the protocol was done by BBA, JT, OAB, TAA, MB, AB, AIA, ORA, HSG, NASA and GO were involved in the. All revisions made to the protocol were approved by those listed in this section.<br>JT and AB, AIA and NASA were international collaborators based outside the region of the study. All the other co-authors including the lead author are based locally in Nigeria.
**b) Clinical study processes:**<br>The Principal Investigator of the IVON trial was BBA and she is based locally in NIgeria. She performed oversight function in the clinical trial with occasional guidance from JT an international collaborator who acted as the clinical trial consultant for this research. HA was the data manager who designed the questionnaire on the REDCap platform supervised by TAA, both of whom are based locally in Nigeria. The IVON trial investigators which comprised all study site coordinators were involved in the data collection at various study sites in Lagos and Kano State. The data collection activities comprised processing blood samples, prescribing medication, patient recruitment, follow up and community tracking. The data collection processes were overseen physically and sometimes remotely by BBA, OAB, TAA, ORA, HSG, RAQ, HA, and AA. The steering committee which comprised BBA, OAB, TAA, MB, ORA, RAQ, and HA conducted quarterly monitoring visits all through the duration of the clinical trial. VOA was an internal monitor for study sites in Lagos State. The second internal monitor is listed as one of the IVON trial investigators because he also assisted in data collection in Kano state where he was based. The internal monitors visit the study sites on a weekly basis to review activities of the clinical trial at each study site.
**c) Data interpretation:**<br>The data generated from the clinical trial was analysed by AIA and BB. They interpreted of the results and shares with the principal and co-investigators. They are both Nigerians, based in the USA.
**d) Manuscript preparation:**<br>The first draft of this manuscript was prepared by OAB, a doctoral student on the IVON trial. This was then reviewed by BBA, the principal investigator and JT, the clinical trial consultant. OAB and ORA

were doctoral students on the IVON trial working on the clinical trial and implementation science aspect respectively. They had as supervisors BBA and other international collaborators, LB, ECL, KSA, CH and AB who participated in the manuscript revision. All other authors also contributed to the manuscription revision. All authors gave approval for the final version of the manuscript to be published. Twenty-two of the 31 co-authors are based in Nigeria.

2. Were the data used in your study collected by authors named on the paper, or have they been extracted from a source such as a national survey? ie, is this a secondary analysis of data that were not collected by the authors of this paper. If the authors of this paper were not involved in data collection, how were data interpreted with sufficient contextual knowledge?

   The Lancet Global Health *believe contextual understanding is crucial for informed data analysis and interpretation.*

   The data for this study were collected by the authors.

3. How was funding used to remunerate and enhance the skills of researchers and institutions based in the area(s) of study? And how was funding used to improve research infrastructure in the area of study?

   Potentially effective investments into long-term skills and opportunities within institutions could include training or mentorship in analytical techniques and manuscript writing, opportunities to lead all or specific aspects of the study, financial remuneration rather than requiring volunteers, and other professional development and educational opportunities.

   Improvements to research infrastructure could be funding of extended trial designs (such as platform trials) and use of master protocols to enable these designs, establishment of long-term contracts for research staff, building research facilities, and local control of funding allocation.

   Skills:
   Most of the site coordinators and nurses employed for this research had no prior experience in clinical trials. The trial gave them the opportunity of being trained in clinical trial methods and practices. They were also trained in basic life support. Some of the co-authors took short online courses from Johns Hopkins University and University of Washington on design and implementation of clinical trials and implementation science respectively. The IVON trial has stimulated the interest of some of the site coordinators who are doctors and the nurses in the research. After the IVON trial, most of them are now involved in other ongoing clinical trials.

   Research infrastructure:
   Through the IVON trial, the health facilities involved in the research were opportuned to have a Hemocue haemoglobinometer for the first time for point-of-care testing for anaemia. Malaria

testing kits and trials drugs were supplied to the sites. Aside these, the sites benefitted from having dedicated fridge/freezer units and a solar system to ensure constant supply of electricity to boost research. In addition, emergency resuscitation equipments like oxygen cylinders, face masks, and instruments for vital signs monitoring were supplied. The research areas were also renovated as requested and chairs and tables for clinical use were purchased for each centre as required to make the researchers and participants more comfortable.

4. How did you safeguard the researchers who implemented the study?

   Please describe how you guaranteed safe working conditions for study staff, including provision of appropriate personal protective equipment, protection from violence, and prevention of overworking.

Personal protective equipment like gloves, nose masks, and nylon aprons were made available especially for use during blood specimen collection and administration of intravenous ferric carboxymaltose. The site coordinators were doctors working in the respective institutions, who were compensated for their time so they could oversee the conduct of the study, supervise the research nurses and ensure the safety of the participants and the quality of the collected data. They were assisted by research nurses, who were employed specifically for the study and carried out the data collection. To prevent overwork, we identified from among the full-time study sites at least one or two more doctors and nurses per study site to assist those employed because some blood specimens have to be taken in the night at times and there are times the research nurses might have more women than expected to deal with.

Benefits to the communities and regions of study

5. How does the study address the research and policy priorities of its location?

   How were the local priorities determined and then used to inform the research question? Who decided which priorities to take forward? Which elements of the study address those priorities?

Nigeria is a country with a high maternal mortality, and the thrust of government and policy makers is to carry out research that can help reduce the maternal and perinatal mortality ratio. Over the years, anaemia has remained among the top ten causes of maternal mortality in Nigeria, contributing to the prevalence both directly and indirectly.

Iron deficiency is the commonest cause of anaemia in pregnancy and the principal investigator, a Professor of Obstetrics and Gynaecology in Lagos, Nigeria, had previously published a study on the prevalence and outcomes of iron deficiency anaemia in her institution. Together with the co-Investigators, most of whom are also based in Nigeria, we decided the priorities to take forward, including the assessment of the prevalence of anaemia, iron deficiency anaemia, preterm birth, depression and breastfeeding practices, amongst others, as we believe that these were important priorities within our context.

We also carried out a formative study where we spoke to pregnant women with and without anaemia, their relatives including husbands and matriarchs, policy makers and health care practitioners. Priorities from these included ensuring the training and re-training of the health workers in administration of intravenous iron, cost of the intervention as well as a desire for clear knowledge of the facts behind the conduct of the trial. We therefore ensured that we included

regular training of the health workers in our methodology, we included cost-effectiveness as one of the outcomes and we created fliers for the participants that clearly illustrated the need and process of the intervention.

As there is a lot of inequity in healthcare even within low-income countries, , we decided to conduct the study in Northern and Southern Nigeria to address issues related to the disparities in both regions. This is because there is a higher burden of poverty and a lower degree of reproductive agency in the Northern compared to the Southern part of Nigeria.

6. How will research products be shared in the community of study?

For instance, will you be providing written or oral layperson summaries for non-academic information sharing? Will study data be made available to institutions in the region(s) of study? The Lancet Global Health *encourages authors to translate the summary (abstract) into relevant languages after paper editing; do you intend to translate your summary?*

Despite the fact that English is the lingua franca in Nigeria, study, we prepared the consent forms in local dialects like Hausa, Igbo, and Yoruba so that less literate participants could understand. We will also share written and oral layperson summaries to all the institutions. We have already prepared a video to this effect, which we plan to translate to the lingua franca of the two regions where the study was carried out.

7. How were individuals, communities, and environments protected from harm?

a) *How did you ensure that sensitive patient data was handled safely and respectfully? Was there any potential for stigma or discrimination against participants arising from any of the procedures or outcomes of the study?*

During the study, the database was only accessible to the Data Manager centrally and it was passworded. The data was de-identified. Confidentiality of the participants' information was ensured. All presentations and publications arising from the IVON manuscript do not contain any sensitive information in any aspect that might compromised the participants' confidentiality. There was no potential for stigma or discrimination against any of the participants as the data collectors were trained properly in appropriate empathy and non-discrimination.

b) *Might any of the tests be experienced as invasive or culturally insensitive?*

Venepuncture was perceived as invasive, and we had a few patients (less than five) refusing to have the procedure at some point during the clinical trial. No aspect of the research was

considered to be culturally sensitive except that in the North we observed that many women had to seek spousal consent before agreeing to participate in the research.

c) *How did you determine that work was sensitive to traditions, restrictions, and considerations of all cultural and religious groups in the study population?*

We took into consideration culture and religious and tried to adapt the research to each setting. A good example is that when we prepared the flyers to be pasted at the study sites to provide information to the women, the first batch prepare with the image of a woman with her hair bare was rejected in the North. We had to design another set of flyers with the same information but with the picture of a woman wearing Hijab (Islamic veil). After a participant withdrew mid-way into the research because the spouse who was previously unaware did not consent, we started encouraging the women in the North to seek spousal consent first before joining. In addition, for community tracking (home visits) we ensured we sent only a female member of the team with whom the participant is familiar, to visit the patient.

d) *Were biowaste and radioactive waste disposed of in accordance with local laws?*

Yes. All the healthcare facilities practice waste segregation and we abided by the rules.

e) *Were any structures built that would have impacted members of the community or the environment (such as handwashing facilities in a public space)? If so, how did you ensure that you had appropriate community buy-in?*

No structure was built for public use in this research. We used existing offices allocated to us by the facility heads for the research.

f) *How might the study have impacted existing health-care resources (such as staff workloads, use of equipment that is typically employed elsewhere, or reallocation of public funds)?*

The Hemocue, fridge/freezers, and solar inverters were donated to the facilities at the end of the IVON trial.

8. Finally, please provide the title (eg, Dr/Prof, Mr/Mrs/Ms/Mx), name, and email address of an author who can be contacted about this statement. This can be the corresponding author.

Name: Prof. Bosede B. Afolabi
Email: bbafolabi@unilag.edu.ng
